# Integrative transcriptome-based drug repurposing in tuberculosis

**DOI:** 10.1101/2025.06.02.657296

**Published:** 2025-06-02

**Authors:** Kewalin Samart, Landon Buskirk, Amy Tonielli, Arjun Krishnan, Janani Ravi

**Affiliations:** 1 Computational Bioscience Program, School of Medicine, University of Colorado Anschutz Medical Campus, Aurora, CO, USA; 2 Data Science, Michigan State University, East Lansing, MI, USA; 3 Biomedical Laboratory Science, Michigan State University, East Lansing, MI, USA; 4 Department of Biomedical Informatics, Center for Health Artificial Intelligence, University of Colorado Anschutz Medical Campus, Aurora, CO, USA.

## Abstract

Tuberculosis (TB) remains the second leading cause of infectious disease mortality worldwide, killing over one million people annually. Rising antibiotic resistance has created an urgent need for host-directed therapeutics (HDTs) — preferably by repurposing existing approved drugs — that modulate host immune responses rather than directly targeting the pathogen. Repurposed therapeutics have been successfully identified for cancer and COVID-19 by finding drugs that reverse disease gene expression patterns (an approach called ‘connectivity scoring’), but this approach remains largely unexplored for bacterial infections like TB. The application of transcriptome-based methods to TB faces significant challenges, including dataset heterogeneity across transcriptomics platforms and biological conditions, uncertainty about optimal scoring methods, and lack of systematic approaches to identify robust disease signatures. Here, we developed an integrative computational workflow combining multiple connectivity scoring methods with consensus disease signature construction and used it to systematically identify FDA-approved drugs as promising TB host-directed therapeutics. Our framework integrates six complementary connectivity methods and constructs weighted consensus signatures from 21 TB gene expression datasets spanning microarray and RNA-seq platforms, diverse cell types, and infection conditions. Our approach prioritized 140 high-confidence drug candidates that consistently reverse TB-associated gene expression changes, successfully recovering known HDTs, including statins (atorvastatin, lovastatin, fluvastatin) and vitamin D receptor agonists (calcitriol). We identified promising novel candidates such as niclosamide and tamoxifen, both recently validated in experimental TB models, and revealed enrichment for therapeutically relevant mechanisms, e.g., cholesterol metabolism inhibition and immune modulation pathways. Network analysis of disease-drug interactions identified 10 key bridging genes (including MYD88, RELA, and CXCR2) that represent potential novel druggable targets for TB host-directed therapy. This work establishes transcriptome-based connectivity mapping as a viable approach for systematic HDT discovery in bacterial infections and provides a robust computational framework applicable to other infectious diseases. Our findings offer immediate opportunities for experimental validation of prioritized drug candidates and mechanistic investigation of identified druggable targets in TB pathogenesis.

## 1. Introduction

Tuberculosis (TB), an infectious disease caused by the bacterial pathogen *Mycobacterium tuberculosis*, is one of the leading causes of millions of deaths worldwide annually [1]. The global rise of antibiotic resistance further impedes the effectiveness of standard antibiotic-based TB treatment [2]. Host-directed therapy (HDT) has been a promising treatment alternative that enhances protective host immune responses against the infection [3] and acts as adjuvant therapy along with antibiotics, often in combination with other HDTs to boost efficacy and minimize resistance [4]. However, identification of HDTs remains a challenge as systemic methods to identify HDT candidates modulating host responses to TB are still lacking.

Drug repurposing enables the identification of potential HDT candidates from the pool of existing FDA-approved drugs, while also reducing the cost and time associated with traditional drug development and making it a suitable strategy for addressing this gap [5]. Computational drug repurposing methods help identify new uses for existing drugs by analyzing large-scale biological data. These approaches can quickly prioritize drug candidates without the need for extensive laboratory screening [6].

Transcriptome-based reversal methods are especially useful because they focus on gene expression—the patterns of genes that are turned on or off in response to disease. A central method in this space is “connectivity mapping”, where disease signatures are computationally matched with drug-induced signatures to find compounds that counteract the disease state to revert it to a healthy state [7]. Early efforts like CMAP 1.0 [8] and LINCS [9] successfully applied this concept to prioritize therapeutics for cancer and neurodegenerative diseases [10], [11], [12].

The COVID-19 pandemic further expanded the utility of these approaches in infectious diseases, with several studies leveraging SARS-CoV-2-induced gene expression changes in infected cells or tissues to identify potential therapeutics using connectivity scores [13], [14], [15], [16] or meta-analysis-based approaches [15], [17], which integrate differential expression results across datasets to generate a set of consensus disease differentially expressed genes. While transcriptome-based HDT repurposing using connectivity scores for infectious diseases has gained significant attention during the COVID-19 pandemic, such approaches have not yet been widely applied to bacterial infections like TB. For instance, recent host-directed therapeutic studies for intracellular pathogens relied on pathway analysis, phenotypic screening, or target-based prioritization without leveraging gene expression reversal schemes [18], [19], [20].

In the context of TB specifically, many studies have applied computational drug repurposing strategies such as meta-analysis, systems pharmacology, molecular docking, ontology-based inference, molecular dynamics simulations, and network-based approaches [21], [22], [23], [24]. While these methods offer mechanistic insights, they typically do not compare the transcriptional host response to TB infection with the host response to drug treatment. As a result, they may overlook compounds that could reverse disease-associated differential expression profiles — a strategy that has shown promise in other disease areas but remains underutilized in TB [25]. Although efforts exist to define robust TB gene signatures [25], these are typically used for diagnostics rather than therapeutic discovery. The limited application of connectivity-based repurposing in TB remains a missed opportunity, given its potential to identify drugs that reverse infection-induced host responses in a data-driven, scalable manner. Despite extensive evidence supporting HDTs for TB in the literature [3], [23], [24], the lack of transcriptome-based drug repurposing applications in this space remains a missed opportunity. Connectivity-based approaches identify drugs that reverse infection-induced host responses. Applying these methods to TB could offer a more systematic and data-driven approach for discovering effective host-directed therapeutics.

Yet, applying transcriptome-based drug repurposing to TB presents key challenges, particularly due to heterogeneity in data and methods:

1. **Lack of a unified scoring framework**: Existing disease-drug signature comparison methods [7] are based on different mathematical principles and often yield inconsistent results. Since no single method is universally optimal, we need integrative or consensus-based frameworks that reconcile outputs from multiple scoring methods, improving robustness and reproducibility in drug repurposing pipelines.
2. **Data heterogeneity in disease signature comparison**: The disease signatures generated from different labs result in a variation in cell/tissue types from primary tissues and cell lines, experimental platforms, infection durations, etc.
3. **Robustness of disease signature**: People respond differently to the same infection, leading to variability in transcriptomic profiles across studies. To identify drugs that are broadly effective, it is essential to extract robust disease signatures that capture conserved differential gene expression changes shared across diverse datasets to reflect core host mechanisms of disease progression.

To navigate the aforementioned challenges, we have developed an integrative host-directed drug repurposing workflow, integrating (i) six connectivity-based approaches categorized into enrichment-based and correlation-based metrics [8], [9], [26], [27] and (ii) a data-driven aggregation of multiple host-response signatures. Here, we apply this new computational framework to TB. Unlike formal meta-analysis, which typically assumes consistent effect sizes and relies on statistical models, our approach integrates diverse transcriptomic datasets spanning different platforms (microarray and RNAseq), infection time points, and tissue or cell types to construct biologically representative consensus disease signatures. This enables us to capture dominant transcriptional responses across heterogeneous studies and improve the robustness of downstream drug reversal predictions. Our novel approach has successfully prioritized potential FDA-approved TB-HDT candidates, including known HDTs such as statins [28], [29], [30] and calcitriol [31] involving TB infection pathways in macrophages. Despite the heterogeneity in disease-drug comparisons and data across biological and technical conditions, our TB HDT predictions show that our integrative method is a promising avenue to identify potential new drug targets. While our primary focus is on transcriptome-based disease-drug reversal, we additionally incorporate network-based analysis for downstream interpretation to uncover mechanistic links between disease genes and known drug targets, identify shared pathways, and prioritize candidate druggable genes based on their topological relevance within the protein–protein interaction (PPI) network demonstrating insights into shared disease-drug pathways for further investigations.

## 2. Materials and Methods

### 2.1 Disease datasets

We began with a representative subset of publicly available TB gene expression datasets that met our inclusion criteria and were available at the time this study was initiated in 2022. Each dataset in our collection includes at least three TB-infected and three healthy control samples, spanning a total of 11 microarray and 10 RNAseq studies (Table S1). We obtained microarray count expression matrices from *refine.bio* [32] and RNAseq count expression matrices from *ARCHS4* [33]. These human gene expression datasets spanned a variety of biological and experimental conditions, such as time points, cell lines, primary tissues, and experimental platforms, providing a practical foundation of data heterogeneity for our analysis.

### 2.2 LINCS drug datasets and signatures

Currently, the LINCS database contains 1.3 million gene expression signatures of drug and small molecule perturbations tested on 3–77 different human cell lines, including six cancer cell lines, all generated using the L1000 assay, which measures the expression of 978 “landmark” genes [9]. LINCS provides drug perturbation data across five distinct levels, each capturing a different aspect of the transcriptional response to drug treatment. These include

- Level 1 (raw data): Unprocessed fluorescence intensity values from the L1000 assay.
- Level 2 (normalized data): Data normalized across samples to correct for technical variation.
- Level 3 (z-scores): Gene expression values standardized to represent deviations from a control condition.
- Level 4 (differential expression signatures): Consensus gene expression changes derived from replicates of a specific condition.
- Level 5 (moderated z-scores): Aggregated gene-level signatures across replicates, summarizing transcriptional effects for each perturbagen.

We used GSE92742 LINCS data level 5, providing drug-perturbation signatures measured on the 978 L1000 genes [9]. Each drug signature is associated with specific experimental conditions: the cell line used for drug treatment, drug concentration in μM, and time point in hours. To ensure consistency and relevance to primary high-throughput compound screens in cell lines [34], we focused exclusively on drug signatures measured at a single concentration (10 µM) and treatment duration (24 h). These specific choices reflect typical screening protocols but also limit the dataset, as not all compounds in LINCS have been tested under these conditions across all cell types. Therefore, we selected a subset of compounds that best met the chosen treatment criteria, resulting in 8,104 compounds, including 728 FDA-approved drugs (reported by Drug Repurposing Hub [35]) uniformly profiled at 10 µM for 24 h across a variable number of cell lines. Only FDA-approved drugs alone yielded a total of 6,093 drug expression signatures across 14 distinct cell lines (A375, A549, ASC, FIBRNPC, HA1E, HCC515, HT29, MCF7, NEU, NPC, PC3, PHH, SKB, VCAP).

### 2.3 Constructing individual disease signatures

We performed differential gene expression (DGE) analysis on microarray datasets using the *limma* package [36], starting from uniformly processed expression matrices and comparing “infected” versus “healthy” samples within each dataset. Similarly, for RNA-seq data, we used the *DESeq2* package [37] to generate up- and downregulated disease gene signatures. For both platforms, individual disease signatures were defined as sets of significantly differentially expressed genes (adjusted *p*-value < 0.05), accompanied by their corresponding log2 fold change (log_2_FC) values. Genes with positive log_2_FC values were classified as upregulated, and those with negative log_2_FC values as downregulated. The full list of disease signatures is provided in Supplementary Table S1. As a result, we obtained 15 upregulated TB-microarray signatures, 12 downregulated TB-microarray signatures, 10 upregulated TB-RNAseq signatures, and 7 downregulated TB-RNAseq signatures. These counts reflect the fact that each study can contain multiple biological conditions, and not every condition yields a gene signature.

### 2.4 Disease signature aggregation

To find a consensus TB signature, aggregated signatures were generated from the individual signatures (Method 2.3) using *the weighted average method* outlined in Figure S1. This aggregation step is based on the assumption that signatures with higher similarity to others are likely to capture more robust and representative disease signals. Therefore, each individual signature was assigned a weight based on its average *Jaccard similarity* to the other TB signatures. The use of the Jaccard index is motivated by the idea that differentially expressed genes consistently shared across multiple signatures are more likely to reflect core disease perturbations and meaningful host responses. Thus, higher similarity scores would imply greater confidence or ‘trust’ and result in higher weights, whereas less similar signatures were down-weighted accordingly.

For a set of n up/down-regulated individual disease signatures derived from a given technology (either microarray or RNA-seq data), we computed gene membership and Jaccard similarity matrices using the following steps.

1. A binary gene membership M is a matrix of size $m\left(\text{genes}\right)\times n\left(\text{signatures}\right)$ where 0 indicates that the gene is not differentially expressed in the signature and 1 otherwise. To capture the magnitude of differential expression, entries of 1 in the matrix were replaced with the corresponding log_2_FC values from the original signatures.
2. We computed a Jaccard similarity matrix J of size n×n, where n is the number of individual disease signatures. Each entry J(i,j) in the matrix represents the *Jaccard* index (see Equation 1) measuring the similarity between the $i^{\text{th}}$ and $j^{\text{th}}$ signatures based on the overlap of their differentially expressed gene sets. Rows and columns correspond to the individual signatures, allowing pairwise comparison of gene content across all signatures.
3. To summarize the overall similarity of an individual signature to other signatures, we computed an average Jaccard vector $V_{j}$ by calculating row-wise mean, resulting in a summarized Jaccard similarity score for each signature. A higher similarity score corresponds to a higher weight in the signature aggregation.
4. The multiplication of the gene membership matrix M and average Jaccard vector $V_{j}$ (Fig. S1; Equation 2) results in aggregated differential expression scores for all genes. These aggregated gene scores reflect how each gene is differentially expressed across all signatures. The final consensus signature (i.e., aggregated signature) only includes landmark genes (to match the gene coverage of the LINCS drug response signatures; Method 2.2) with absolute aggregated scores greater than 0.4 (i.e., genes that are highly differentially expressed in at least 40% of the individual signatures (Equations 3 and 4).

Equation 1. $$\text{Jaccard}\;\;\text{score}\left(S_{i},S_{j}\right)=\frac{\left|S_{i}\cap S_{j}\right|}{\left|S_{i}\cup S_{j}\right|}$$

where $\left(S_{i},S_{j}\right)$ is a pair of differentially expressed gene sets of the signatures $S_{i}$, $S_{j}$.


Equation 2. $$V_{aggr_{up/dn}}=V_{J_{up/dn}}\times M_{up/dn}$$

where $V_{aggr_{up/dn}}$ is an aggregated disease vector (in either upregulated or downregulated directions) dimension of 978 × 1 filled with aggregated gene scores, where 978 corresponds to the number of landmark genes.

$V_{J_{up/dn}}$ is an average Jaccard vector;

$$S_{aggr_{up}}=V_{aggr_{up}}\left(aggregated\;gene\;scores>0.4\right)$$

Equation 4. $$S_{aggr_{dn}}=V_{aggr_{dn}}\left(aggregated\;gene\;scores<-\;0.4\right)$$

### 2.5 Quantifying potential candidate drugs using multiple scoring methods

The core principle of transcriptome-based drug repurposing is that an efficacious drug candidate should reverse the disease signature patterns (i.e., show negative connectivity). In the past decades, many connectivity methodologies [8], [9], [26], [38], [39], [40], [41] have been developed to quantify disease-drug relationships based on their reversal effects. Given these different existing methods developed upon each other, they could be broadly categorized into two groups based on disease-drug similarity metrics used: (i) enrichment-score-based (ES-based) methods and correlation-based (i.e., pairwise-similarity) methods [7].

#### 2.5.1. Enrichment-score-based methods

Given a pair of disease and drug signatures, ES-based connectivity methods take up- and downregulated disease gene sets and the ranked drug gene list to compute a disease-drug connectivity score built on the enrichment score calculation adapted from Gene Set Enrichment Analysis (GSEA): a more negative score indicates stronger reversal of the disease signature by the drug [42]. This method captures both the distribution and membership of up- and down-regulated disease genes appearing on drug profiles [7], [8], [42].

We included four ES-based methods in our analysis.

1. **CMAP 1.0 Connectivity Score (CS)** calculates a disease-drug enrichment score using a signed one-sample Kolmogorov–Smirnov (KS) test, which compares the empirical distribution of disease gene positions in the ranked drug gene list to a reference uniform distribution [7], [8].
2. **All three CMAP 2.0 (LINCS) scores** extend this approach using a weighted, signed two-sample KS statistic, directly adapted from GSEA, to evaluate how strongly disease genes are enriched at the extremes of a drug signature [7], [9]. Within this scoring system, the **Weighted Connectivity Score (WCS)** provides a nonparametric measure of disease-drug similarity. The **Normalized Connectivity Score (NCS)** rescales the WCS to account for variability across cell lines and drug types, enabling direct comparisons. Finally, the **Tau score (τ)** further adjusts the NCS by contextualizing it within a reference distribution of connectivity scores, thereby correcting for nonspecific associations and highlighting more selective disease-drug connections.

#### 2.5.2. Pairwise-similarity-based methods

Another type of connectivity method is based on pairwise-similarity metrics. These correlation-based approaches capture the degree and direction of similarity between the expression patterns of disease and drug; a negative correlation suggests a potential reversal effect. Here, we implemented two of them, denoted as XCor (extreme Pearson) and XSpe (extreme Spearmen) [7], [26]. These two metrics are based on the correlation between two ranked sets of values, in our case, differential gene expression values (log_2_FC) for a pair of disease and drug signatures.

1. **XCor** calculates the Pearson correlation between log_2_FC values of significantly differentially expressed genes (adjusted p-value < 0.05) shared between the disease and drug signatures.
2. **XSpe**, on the other hand, computes the Spearman rank correlation, comparing the ranks of log_2_FC values for overlapping significantly differentially expressed genes in the disease and drug signatures.

### 2.6 Finalizing top-rated drug candidates

To cope with the heterogeneity in drug data stemming from multiple cell lines, drug concentrations, and time points, we quantify the potential drugs based on the drug reversal score across cell lines restricted to only one drug condition with the 24-hour time point and concentration of 10 μM across drugs since this is a primary drug condition present in the initial LINCS perturbation high-throughput screens of cell lines (Method 2.2) [34]. For each drug signature associated with a cell line, we calculated the median reversal score across multiple cell lines to summarize the overall reversal independent of the cell line variability. This cell line-agnostic score was then used in downstream drug prioritization.

#### 2.6.1 Selections of drugs reversing the majority of individual disease signatures

To identify strong drug candidates, we prioritized those that consistently showed negative connectivity scores to most of the individual disease signatures, indicating potential to reverse disease-associated differential expression patterns. For each connectivity method, we selected only those drugs that were ranked in the top 10% most negative median reversal scores and reversed at least 50% of the individual disease signatures. Since we performed drug prioritization with six connectivity metrics, six prioritized drug lists were obtained; one list per metric: CMAP: (i) CS, LINCS: (ii) WCS, (iii) NCS, (iv) Tau (т), and pairwise-similarity-based: (v) XCor, (vi) XSpe.

#### 2.6.2 Selections of drugs reversing aggregated signatures

To prioritize drugs based on the aggregated disease signatures, we selected the top 10% of drugs with the most negative connectivity scores, indicating strong potential to reverse the aggregated disease-associated differential expression patterns. As in the previous section, this was done separately for each of the six connectivity methods.

#### 2.6.3 Method-wise filtering approach and rank aggregation

All analyses described above, from disease signature construction to drug prioritization using each of the six connectivity metrics, were performed independently for microarray and RNA-seq datasets, and separately for individual and aggregated disease signatures. This resulted in four separate drug prioritization pipelines: (i) individual microarray signatures, (ii) aggregated microarray signatures, (iii) individual RNAseq signatures, and (iv) aggregated RNAseq signatures. We next sought to consolidate these results by performing a method-wise filtering and rank aggregation within each pipeline to identify robust top candidates.

**Figure 1.**
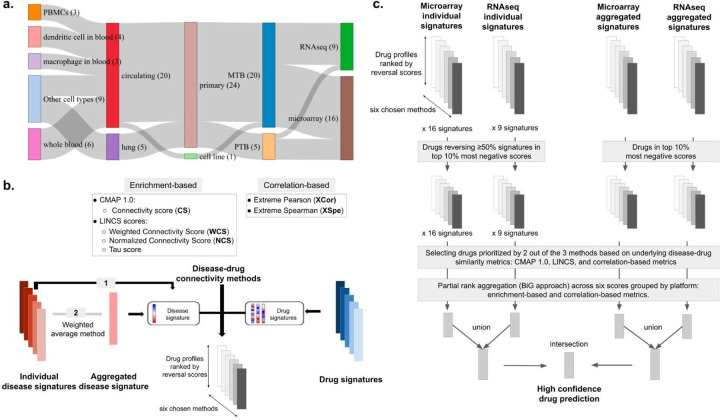
Overview of disease signature datasets and the integrative drug repurposing workflow. **(a)** Sankey diagram summarizing the biological and technical heterogeneity across the TB transcriptomic signatures. These signatures were constructed from multiple TB datasets spanning diverse biological sources, including different tissue and cell types, sample origins (circulating blood vs. lung), sample types (primary vs. cell line), TB types (PTB: pulmonary TB vs. MTB: active TB cases without explicit “pulmonary” specification), and profiling technologies (microarray and RNA-seq). **(b)** Overview of our integrative drug repurposing workflow implementing (i) individual and (ii) aggregated signatures compared against drug signatures using six connectivity scoring methods: CMAP 1.0 and LINCS scores (WCS, NCS, Tau) for enrichment-based, and Extreme Pearson (XCor) and Spearman (XSpe) for correlation-based approaches. **(c)** Drug prioritization pipeline applied to microarray and RNA-seq signatures, including both individual and aggregated versions. Drugs reversing ≥50% of the disease signature with scores in the top 10% most negative values were selected, and only those supported by ≥2 of 3 metric subgroups (CMAP 1.0, LINCS, correlation-based) were retained. Final drug rankings were generated using Bayesian Latent Variable Approach for partial rank aggregation; “BiG” method [44], and high-confidence predictions were obtained by intersecting results across signature types.

To ensure that the drug prediction results are robust across different connectivity measures, the drugs appearing only once out of the three method categories (CMAP, LINCS, and correlation-based) were excluded. Consequently, analyzing results from microarray and RNAseq separately, the remaining drugs in the lists from both individual and aggregated disease signatures were then summarized into two drug lists (one per signature type) using the rank aggregation method ‘BiG’ [43], [44] (treating missing genes as bottom-ranked genes). We chose this rank aggregation approach according to Wang et al. as it provides robust aggregation performance regardless of heterogeneity in method effectiveness across the input lists [43]. Here, we aggregated drug lists using two heterogeneity sources which were the two different mathematical concepts behind each connectivity score: (i) ES-based, and (ii) correlation-based (Fig. 1c). This step resulted in four rank-aggregated drug lists including microarray and RNAseq predicted drug lists from individual signatures, and from aggregated signatures.

#### 2.6.4 Finalization of highly confident predicted drugs

To identify a robust set of candidate drugs, we first took the union of microarray and RNA-seq rank-aggregated results (each derived across multiple connectivity metrics) separately for the individual and aggregated TB signatures. We then computed the intersection between these two sets. This process yielded a final list of 140 high-confidence drug candidates (Table S2) that were consistently prioritized using both (i) individual and (ii) aggregated TB gene signatures.

### 2.7. Disease pathway enrichment analysis

To delineate perturbed pathways underlying TB, we performed pathway enrichment analysis on the individual disease signatures from both microarray and RNAseq (from 2.3 and 2.4) to identify commonly enriched Gene Ontology Biological Process (GO:BP) terms [45]. The enriched GO:BP terms were identified using the *ClusterProfiler* R package [46], where genes annotated to GO terms that are present in any of our disease datasets were used as the background genes. Only GO:BP terms with at least 5 and at most 200 annotated genes were included for downstream tests. The selected GO:BP terms have a *p*-value cutoff of <0.05, an adjusted *p*-value (*q*-value) cutoff of < 0.1 corrected for multiple hypothesis testing with Benjamini-Hochberg procedure.

#### 2.7.1 Pathway cluster analysis

A pathway enrichment analysis was performed on each individual disease signature, independently; then the union of all enriched GO:BP terms across all disease signatures was grouped into clusters based on functional similarity using a binary cut method via *SimplifyEnrichment* [47]. The binary cut approach groups terms that are semantically similar to each other into the same cluster. The top three GO:BP terms, the pathways associated with the highest FDR-adjusted statistical significance values, i.e., *q*-values in each cluster, were selected to be the cluster representatives of enriched pathways.

#### 2.7.2 Biological alignment between the aggregated and individual signatures at the pathway and pathway cluster levels

**Figure 2.**
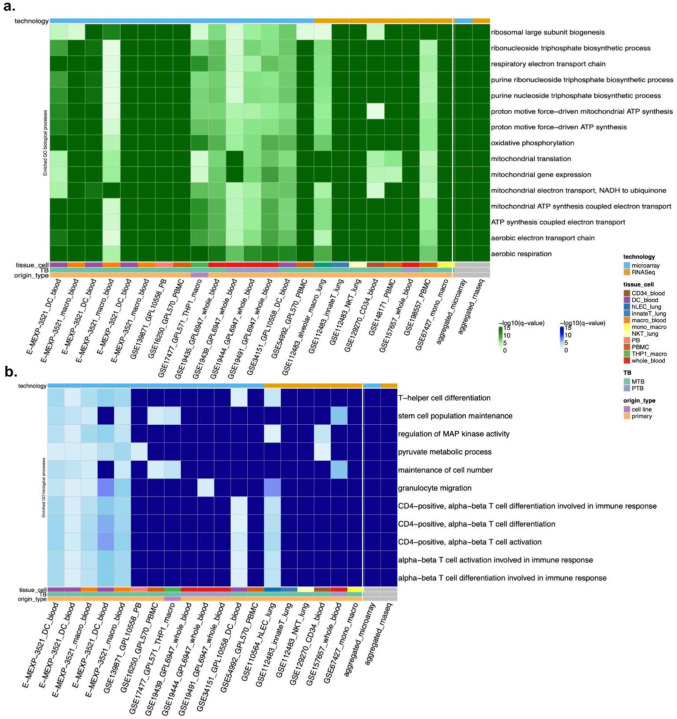
Aggregated signatures capture highly variable pathways across individual signatures. **(a)** Heatmap showing highly variable GO biological processes (GO:BP) across the individual TB transcriptomic signatures that are enriched in the aggregated upregulated TB signature. Many of these pathways are consistently enriched across studies, reflecting host metabolic adaptation and mitochondrial stress during TB infection. **(b)** Heatmap showing highly variable GO:BP terms across individual TB signatures that are enriched in the aggregated downregulated TB signature. These pathways include key immune-related processes such as T-helper cell differentiation, CD4-positive T-cell activation, and MAP kinase signaling. Signature annotations (profiling technology, tissue/cell type, TB sample annotation (PTB: pulmonary vs. MTB: non-specified), and origin type) are indicated in the annotation bars, highlighting the biological and technical diversity across datasets.

To evaluate how well the aggregated disease signatures reflect the biological signals captured in individual signatures, we performed three complementary analyses:

1. **Cluster coverage analysis**: We computed the pathway clusters enriched in the individual signatures and examined whether the aggregated signatures also showed enrichment within those same clusters. This assessed whether all key biology from individual analyses was preserved in the aggregated representation (Figure S2).
2. **Conservation of highly variable pathways**: We identified pathways that were consistently enriched across a majority of individual signatures (defined as being enriched in at least 30–50% of the datasets, depending on observed variability) and assessed their presence in the aggregated enrichment results. These “highly variable” pathways serve as a proxy for robust, cross-study biological signals. This comparison demonstrates that aggregation preserves both widespread and functionally coherent disease-related signals (Figure 2).

### 2.8 Statistical tests to evaluate the effects of confounding variables in disease pathway cluster enrichment

To investigate whether observed differences in pathway enrichment could be associated with specific study-level metadata rather than true disease signals, we followed the cluster coverage analysis (Methods 2.7.2) result with statistical tests across multiple metadata categories. Specifically, we evaluated whether clusters of enriched GO:BP terms varied significantly across: (i) tissue/cell type, (ii) tissue origin: circulating vs. lung, (iii) disease subtype: pulmonary TB (PTB) vs. extrapulmonary/unspecified TB (MTB), (iv) microarray platform (e.g., GPL570, GPL10558) and (v) profiling technology: microarray vs. RNAseq.

Each individual disease signature was associated with its enriched GO:BP terms and assigned to pathway clusters as described in Methods 2.7. For each metadata category, we conducted a Mann–Whitney U test to assess whether the distribution of enrichment scores (–log10(q-value)) for each pathway cluster differed significantly between metadata-specific groups (e.g., PTB vs. MTB). This analysis enables the identification of pathway clusters whose enrichment patterns may be confounded or driven by metadata variation, rather than representing consistent disease biology. Results are discussed in Results 3.2–3.3.

### 2.9 Constructing cholesterol- and vitamin D-related disease-drug pathway subnetworks

To investigate shared biological mechanisms between predicted HDTs and TB-related disease genes, we constructed two pathway-relevant subnetworks focused on cholesterol metabolism and vitamin D signaling in human macrophages. These pathways were selected based on the frequent occurrence of HMGCR inhibitors (statins) and vitamin D receptor agonists (calcitriol) that are well known among the top-ranked predicted drugs.

We first selected prioritized drugs annotated with either HMGCR inhibition or vitamin D receptor agonism as their MOAs. For each drug, we included its known protein targets with interaction scores in the top 90^th^ percentile obtained from the Drug–Gene Interaction Database (DGIdb) [48], and the top and bottom five most perturbed genes based on GSE92742 LINCS level 5 expression data (24-hour, 10 μM conditions) [9]. Drugs were linked based on shared enriched pathways identified via Drug Set Enrichment Analysis (DSEA) implemented in the *signatureSearch* package. To expand each subnetwork, we integrated high-confidence inferred protein–protein interactions (confidence score ≥ 0.7) from the STRING database. Finally, we intersected each subnetwork with aggregated TB disease gene signatures to identify shared components between disease-related genes and drug-associated molecular effects.

This approach enabled systematic mapping of candidate HDT drugs and disease-associated genes onto biologically relevant cholesterol and vitamin D pathways, initially providing a network-based framework to explore shared host response mechanisms in TB.

### 2.10 Building disease-drug gene/pathway networks

To explore potential mechanistic links between TB disease genes and predicted drug targets, we constructed disease-gene/pathway-drug networks using the STRING protein-protein interaction (STRING-PPI) network. The STRING-PPI network consists of interactions (edges) across ~23k human genes (nodes) [49]. The disease-associated genes were derived from our aggregated TB signature, combining upregulated and downregulated genes that consistently appear in both microarray and RNAseq datasets (resulting in a total of 7 disease genes defined as *source nodes*). Known drug target genes (i.e., target nodes) for each predicted drug were obtained from DGIdb [48], selecting only those targets with interaction scores in the top 90^th^ percentile. For each disease gene/drug target gene pair, the shortest path within the STRING network was calculated using the average Betweenness Centrality (BC) score for all nodes along the path. The BC score quantifies the importance of a gene in facilitating information flow in the PPI network, allowing us to prioritize genes that are likely to serve as key intermediates between disease pathways and drug mechanisms of action. We selected the top 2% of paths with the highest average BC scores per each source-target gene pair, resulting in 10 key in-path genes potentially mediating disease-drug interactions.

### 2.11 Investigating potential drug mechanism of action pairs showing synergistic and antagonistic effects

Synergy between mechanisms of action (MOAs) is key to designing effective host-directed combination therapies for TB, as it can enhance efficacy by targeting complementary biological processes. To characterize potentially synergistic effects of key MOAs associated with our predicted TB HDT candidates, we first took the union of the prioritized drug sets from both microarray and RNAseq TB datasets and identified their corresponding MOAs. For each drug pair among the predicted candidates, we retrieved corresponding synergy scores from *DrugCombDB* (retrieved on 07/24/2024) [50], including ZIP [51], Bliss [52], Loewe [53], and HSA [54] scores. To combine these four synergy scores, we computed z-scores for each metric across all available drug pairs listed in *DrugCombDB* and calculated a combined z-score by taking the average for each drug pair.

These combined z-scores represent the overall synergy and antagonism strength for each drug pair. We then mapped each drug present in our union drug set to its associated MOAs and summarized the average combined z-score across all drug pairs for each unique MOA–MOA combination. This approach enabled us to incorporate all MOAs represented in the predicted drug set and evaluate potential functional synergy or antagonism between them. The MOA synergy heatmap (Figure 4) was generated to visualize synergy patterns across MOAs and highlight MOA pairs with strong evidence of synergy or antagonism based on combined z-scores.

This analysis enabled systematic evaluation of both synergistic and antagonistic relationships between drug mechanisms, informing rational selection of complementary or avoidable MOA combinations for potential TB HDT combinations.

### 2.12 Baseline comparison

While drug mechanism synergy provides insight into potential combinatorial efficacy, assessing how well disease and drug signatures align at baseline tissue types is equally critical for meaningful reversal predictions. To address this, we evaluated the similarity between healthy control disease samples and untreated drug cell line profiles to identify the most biologically appropriate reference cell types for disease-drug comparison.

We obtained drug baseline samples from L1000 Level 3 profiles and preprocessed them to retain only 24-hour treatment samples to be consistent with the primary analysis. Quantile normalization was applied to each sample (to ensure consistent expression distributions). To form cell line profiles, these drug baseline samples were aggregated across each cell line using either the mean or Stouffer-like weighted mean (pairwise correlations among replicates to derive weights that emphasize internally consistent samples). The control disease samples were sourced from microarray and RNA-seq datasets, filtered to match the landmark genes profiled in the cell line data, and quantile normalized relative to the drug control data.

We applied four correlation metrics: Pearson, Spearman, Rank-biased overlap (RBO), and Lasso regression coefficients to quantify similarity between control samples and cell lines. These metrics were selected to capture different aspects of concordance of gene expression values. While Pearson and Spearman measure linear and monotonic associations, respectively, RBO emphasizes top-ranked gene agreement. In addition, Lasso regression incorporates sparsity to highlight a subset of predictive gene–gene relationships. These correlation matrices were computed between all cell lines and control samples for each selected correlation type.

## 3. Results

### 3.1 Systematic prediction of host-directed therapeutics from TB transcriptomes

Effective drug repurposing for TB (or any disease with heterogeneous signatures) requires approaches that can handle biological and technical variability across transcriptomic datasets while capturing meaningful disease signatures for targeted therapeutic discovery. To mitigate challenges arising from transcriptomic data heterogeneity and differences in disease-drug comparison methods, we developed an integrative computational workflow that integrates six connectivity-based methods to prioritize drugs: individually and via consensus disease signature construction (Figure 1b; Methods 2.5). Our approach was designed with consideration of three major sources of heterogeneity: (i) two data generation technologies (microarray and RNAseq), (ii) various biological conditions (cell types, tissues, time points), and (iii) unresolved best metrics for disease-drug comparison.

We first generated individual TB disease signatures starting with uniformly processed gene expression datasets from 11 microarray and 10 RNAseq studies, resulting in 25 signatures: 16 microarray and 9 RNAseq signatures (Figure 1a; Methods 2.1–2.2; Table S1). Individual disease signatures were constructed via differential gene expression analysis, capturing significantly up- and down-regulated genes per contrast. To construct robust consensus signatures representing dominant TB responses, we aggregated the individual signatures using a weighted Jaccard similarity approach (Methods 2.3), prioritizing genes significantly and consistently perturbed across datasets.

We obtained drug perturbation profiles from the LINCS L1000 dataset (Methods 2.4) and implemented the six connectivity scoring scores, which use four enrichment-based (CMAP: CS, LINCS: WCS, NCS, Tau) and two correlation-based (Extreme Pearson, Extreme Spearman) methods to quantify disease-drug reversal potential (Methods 2.5). To minimize variability across conditions of drug data, we only utilized drug signatures with a concentration of 10 µM and treatment duration of 24 hours, followed by computing median reversal scores across cell lines (varying between 1–14) per drug (Methods 2.6).

For individual TB signatures, drugs were prioritized based on their ability to reverse a majority of the input TB signatures (at least 50% of signatures); the top 10% drugs (highest negative scores) were selected (Methods 2.6.1). Drug predictions from aggregated signatures were defined as the top 10% of drugs with the most negative disease-reversal scores (Methods 2.6.2). These drug predictions from individual and aggregated signatures across all the six connectivity metrics were categorized into three method types: CMAP 1.0, LINCS, and correlation-based methods. We selected drugs that consistently appeared in at least two out of the three method types. Then, all these drug lists across methods were combined using a robust rank aggregation approach [44] (Methods 2.6.3). This combinatorial approach yielded highly confident drug candidate lists for microarray and RNAseq signatures, analyzed separately. These two lists were subsequently combined (union) to enable further downstream biological interpretation through network-based analyses (Methods 2.6.4). At last, we intersected these two lists to derive the final high-confidence drug predictions.

In summary, we developed an integrative workflow that enabled systematic prioritization of candidate HDTs for TB by incorporating heterogeneous transcriptomic datasets, and multiple disease-drug connectivity scoring methods. By combining results from both individual and aggregated consensus TB signatures across microarray and RNA-seq platforms, we generated high-confidence drug candidate lists that are well-suited for further mechanistic interpretation and validation.

### 3.2 Time-dependent sampling in a microarray study reveals unique transcriptional features

After constructing individual and aggregate TB signatures, we next investigated the functional relevance of these gene signatures by examining perturbed biological pathways. Given the heterogeneity among TB signatures from different studies, platforms, and biological contexts, we aimed to identify key factors contributing to variation in underlying disease mechanisms. We clustered enriched GO:BP (Gene Ontology, Biological Process) terms from both individual and aggregated TB signatures and observed significant variance across studies (Methods 2.7.1; Figure S2). These pathway clusters reflect biological mechanisms captured by TB signatures from both microarray and RNAseq. Notably, variation in gene expression profiling technology, microarray versus RNAseq, did not account for the observed pathway clustering, as no GO:BP terms were significantly different between the two technologies based on the Mann-Whitney U test (Methods 2.8). Instead, clustering patterns were primarily driven by either biological or platform differences between upregulated signatures derived from a particular profiling study, E-MEXP-3521, from human dendritic cells and macrophages infected with *Mycobacterium tuberculosis* across multiple time points (4, 18, 48 hours), and those from other microarray experimental platforms of other microarray studies (Figure S1).

To clarify whether the observed differences arose from biological context or platform-specific effects, we compared the pathway enrichment profiles of each individual microarray platform against all others. The assumption was that if the variation were driven by biological context, the differentiating pathways should not simply mirror technical aspects of the study design. Conversely, if platform-specific effects were responsible, other microarray platforms would also show significant differences, and those differences would be consistent across comparisons.

Among all comparisons, only E-MEXP-3521 showed statistically significant differentiating GO:BP terms, suggesting distinct transcriptional patterns compared to other individual signatures. The most enriched biological processes for E-MEXP-3521 involve the nucleus and structures called Cajal bodies and telomere regions which help maintain nuclear architecture and genomic function. We also found signals related to how the cell controls the location of certain RNA molecules and proteins. These patterns may reflect how the cell reorganizes its internal structure and gene activity over time during TB infection. In addition to these nuclear-related processes, E-MEXP-3521 also showed unique enrichment for pathways involved in proton transmembrane transport, regulation of superoxide anion generation, and NADP metabolic processes, revealing oxidative stress response and metabolic reprogramming over time.

We also performed the same statistical tests across other confounding factors, including tissue/cell type, PTB vs. MTB, and tissue origin (circulating vs. lung), but did not find any significant differences in GO:BP enrichment. While the comparison between cell line and primary samples revealed some statistically significant GO:BP terms, the results lacked sufficient statistical power to draw a confident conclusion. This confirms that, although our TB signatures are heterogeneous overall, E-MEXP-3521 was the only dataset that showed a distinct transcriptional profile with statistically significant pathway differences, suggesting that TB signatures have more common ground on average. Since this study was uniquely sampled across multiple time points and the enriched terms are related to nuclear structure and oxidative stress, the variation is likely driven by temporal regulation rather than platform-specific effects.

### 3.3 Biological pathways associated with the aggregated signatures are enriched across the individual signatures

Given the heterogeneity observed among individual TB signatures and the lack of statistically significant clustering by biological or technical conditions (Result 3.2), we next asked whether aggregating these signatures could help summarize dominant, biologically relevant disease signals. To this end, we combined the individual signatures into one aggregated signature for up- and down-regulated directions, independently, using a weighted average approach with *Jaccard* similarity index as a proxy for ‘trust’ (Method 2.3). An individual signature was given more weight (i.e., more trust) if it was more similar to the rest of the individual signatures in the set, and less weight otherwise. The goal is to capture dominant disease signals by amplifying consistent patterns, which are presumably biologically relevant signals, while downweighting signals from potentially confounding variables associated with individual signatures, such as experimental platform, tissue/cell types, and infection time points (Method 2.3).

To evaluate whether the aggregated signature successfully recapitulates the major biological processes underlying TB as observed in individual signatures, we first examined the coverage of highly variable GO:BP pathways enriched across individual datasets (Methods 2.7.2). We found that all the highly variable pathways enriched in the aggregated signatures were also broadly enriched in the individual signatures (Figure 2). These conserved pathways are primarily involved in mitochondrial and cellular energy-related functions, including mitochondrial gene expression and translation, oxidative phosphorylation, ATP synthesis, aerobic respiration, and electron transport chain activity. Additionally, nucleotide biosynthesis and ribosomal biogenesis pathways were also consistently enriched.

We observed a similar trend at the level of pathway clusters, which revealed broad enrichment of key immune and stress-related processes, including MAPK and STAT signaling, interleukin-1 response, and cell killing; mitochondrial and nuclear transport functions; vascular and smooth muscle remodeling; erythrocyte development and homeostasis; collagen metabolism; and nitric oxide biosynthesis. These results indicate that the aggregated signature effectively summarizes the recurring immune-metabolic and stress response themes that are characteristic of TB-related host perturbation.

Overall, this demonstrates that the pathways identified from the aggregated signatures are consistently enriched across individual signatures, particularly for immune, metabolic, and stress-adaptive processes. This observation is further supported by a Mann–Whitney U test, which found no statistically significant differences in GO:BP term enrichment between aggregated and individual signatures, which indicates that aggregation effectively captures the underlying disease biology shared across studies.

### 3.4 Predicted candidate HDTs with literature evidence against TB

Building on the prioritized drug lists from our integrative transcriptome-based workflow, we next asked whether the top predicted candidates include drugs with known or emerging HDTs with potential against TB. This analysis aims to assess the biological relevance of our predictions and provide external corroboration from published literature.

Our workflow successfully prioritized well-known potential HDTs for TB, such as calcitriol [31] and statins [28], [30], [30], including atorvastatin [55], fluvastatin [56], lovastatin [57], and rosuvastatin [58]. Further, Niclosamide, the third highest ranked of predicted drugs, has been recently validated as a TB therapeutic candidate. It induces respiratory stress and disrupts fatty acid metabolism, suggesting a novel mode of action [59]. Additionally, the drug candidate tamoxifen has shown activities against TB infection both *in vivo* and *in vitro* by enhancing lysosomal activity and increasing the delivery of mycobacteria to lysosomes, resulting in reduced bacterial survival, in both cell and animal models [4] (Table 1).

Importantly, the top predicted drug candidates are associated with distinct MOAs, including HMGCR inhibitors, i.e., statins and vitamin D receptor agonists, frequently across microarray and RNAseq TB datasets (Table 2 and Section 3.5). We also investigated the underlying molecular mechanisms of these 25 TB signatures to better understand potential technical and biological signals driving our predictions.

### 3.5 Exploring well-known shared disease-drug biological pathways: cholesterol- and vitamin-D-related pathways

Based on the MOAs of our predicted HDT candidates, we further investigated two well-characterized and frequently implicated pathways: cholesterol metabolism and vitamin D signaling. These pathways are supported by prior evidence of their involvement in host responses to *M. tuberculosis*, particularly within macrophages [78].

To explore how our predicted drugs and disease genes converge on these biological processes, we constructed two separate subnetworks: one for cholesterol-related and one for vitamin D-related pathways (Figure S3). Each subnetwork was initiated from prioritized drugs associated with HMGCR inhibitors or vitamin D receptor agonists. We then incorporated known targets of the drugs as well as drug-perturbed genes (top/bottom 5 differentially expressed genes from LINCS drug signatures). Connections between drugs were added based on shared pathway enrichment using DSEA (Methods 2.9), and networks were expanded with high-confidence inferred interactions from the STRING protein–protein interaction (PPI) database.

We observed that, in the cholesterol-related subnetwork, key genes such as LIPA, BHLHE40, and HES1 appeared as both disease-associated and drug-targeted genes. In the vitamin D-related subnetwork, BHLHE40 was the sole overlapping gene between the disease signature and drug perturbation network. These findings highlight biologically meaningful intersections between our predicted HDTs and disease mechanisms, supporting the therapeutic relevance of targeting these pathways in TB, potentially via these common disease-drug genes (Figure S3).

### 3.6 Identified novel druggable gene targets and highly confident potential TB HDT candidates via a network analysis

**Figure 3.**
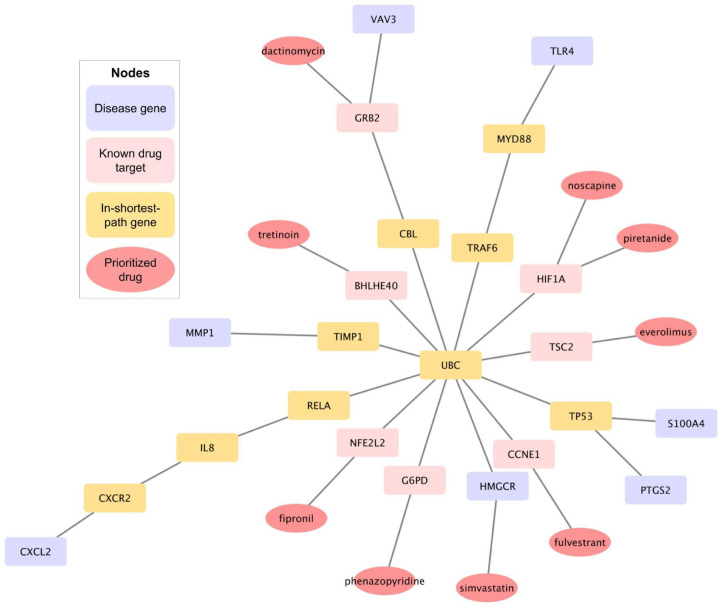
Disease-drug-target network identifies key linking genes and prioritized drug candidates. Network visualization showing the shortest-path connections between TB disease genes (purple), known drug targets (pink), and prioritized drugs (red) through intermediate in-path genes (yellow) within the STRING genome-wide host protein-protein interaction network. This analysis highlights 10 key in-path genes—including MYD88, RELA, CXCR2, UBC, GRB2, CBL, TIMP1, TRAF6, IL8, and TP53—that serve as mechanistic bridges between TB-perturbed genes in our aggregated signatures and known drug targets. Prioritized drug candidates (red nodes) connected through these paths include both known TB-relevant HDTs such as simvastatin, fulvestrant, and novel predictions such as fipronil, phenazopyridine, tretinoin, dactinomycin, noscapine, piretanide, and everolimus. These network-level insights support the biological relevance of our drug predictions and suggest new avenues for host-directed TB therapeutics.

While our integrative transcriptome-based drug prioritization relies on global reversal patterns between disease and drug signatures, network analysis provides additional gene-level insights by uncovering linking genes serving as bridges or paths between disease-perturbed genes and known drug targets within the genome-wide gene network. These in-path genes are mechanistic hints showing how disease mechanisms and drug actions can cross within host pathways, potentially revealing new therapeutic targets. Using the approach described in Methods 2.10, we identified 10 key in-path genes linking TB disease genes and drug targets, including MYD88, RELA, CXCR2, UBC, GRB2, CBL, TIMP1, TRAF6, IL8, TP53 (Figure 3; Table 3). By selecting only those with known target genes that form the strongest connections (highest average betweenness centrality, BC scores) with the TB disease genes in the network, we further narrowed to 9 highly confident drugs from our final list of 140 prioritized drugs (candidates overlapping between individual and aggregated signatures’ predictions). These highly confident drugs include both well-known potential TB HDTs, such as simvastatin and fulvestrant, and less-recognized candidates that may represent novel therapeutic opportunities based on their high network proximity to TB disease perturbation genes. These novel candidates include fipronil, phenazopyridine, tretinoin, dactinomycin, noscapine, piretanide, and everolimus (Figure 3). These results further support our predictions, since the network analysis not only recovers known TB-related genes among the in-path genes but also reveals novel ones, strengthening the biological relevance of the identified drug candidates.

### 3.7 Potential synergistic MOA combinations against TB from our drug predictions

**Figure 4.**
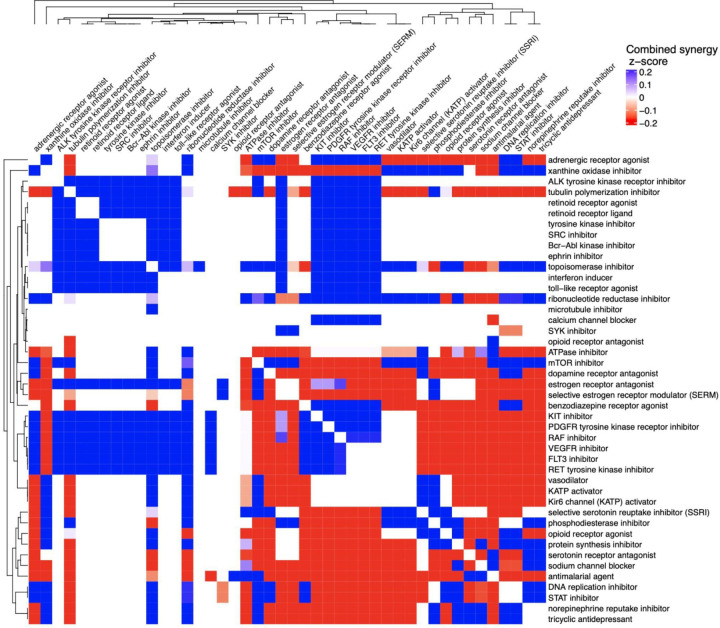
Synergy and antagonism of pairs of mechanisms of action (MOA) associated with our 140 high-confidence drug candidates. The heatmap shows combined synergy Z-scores for all pairwise combinations of MOAs derived from our predicted TB drug candidates. Synergy scores were computed by integrating four synergy metrics (ZIP, Bliss, Loewe, and HSA) from *DrugCombDB* and summarized using combined Z-scores (blue = synergy; red = antagonism).

Following our strong prioritized list of TB drug candidates, as a final piece, we investigated which of these drugs can function effectively in synergistic combinations against TB infection. For each candidate drug pair, we computed synergy scores using four standard synergy metrics and mapped the results to their corresponding MOAs to summarize overall synergy across all unique MOA pairs (Methods 2.11). Among the top synergistic MOA pairs computed (Figure 4), we observed literature-supported synergy between kinase inhibitors (e.g., RAF, VEGFR, FLT3, ALK, RET) and tubulin polymerization inhibitors, indicating that dual targeting of proliferative and cytoskeletal pathways may enhance efficacy when used together [90]. Retinoid receptor agonists and ligands showed cooperative activation effects on immune-related gene expression, suggesting possible synergy in modulating host transcriptional programs [91]. Combinations involving broad tyrosine kinase inhibitors and SRC inhibitors, or Bcr-Abl and ephrin pathway inhibitors, are supported by multi-target drugs like dasatinib, which exhibit activity across these pathways [92], [93].

In contrast, several MOA combinations were identified as antagonistic in our analysis, particularly those involving SSRIs, sodium channel blockers, phosphodiesterase inhibitors, opioid receptor agonists, tricyclic antidepressants, or serotonin receptor antagonists when paired with kinase inhibitors. While these pairs showed negative synergy scores, we did not find obvious existing evidence that these MOAs truly are agnostic with one another. The observed antagonism may come from how these drugs act on different or same systems. For instance, SSRIs or opioid receptor agonists can weaken immune signaling, which might counteract the effects of kinase inhibitors [94]. In addition, both sodium channel blockers and SSRIs, which are drug classes that showed antagonistic interactions in our analysis, can affect heart rhythm, and their combined use may increase the risk of cardiac side effects [95], [96]. This suggests that anyone on these long-term medications should avoid the antagonistic drug candidates, further adding value to these top predictions. These interactions may also depend on factors like dose, timing, or cell type. Further experimental studies are needed to determine whether these combinations should be avoided.

Together, this analysis provides a guide for prioritizing synergistic drug mechanism combinations and avoiding antagonistic interactions (especially when other drugs are being taken for comorbidities) in the context of TB HDTs.

## 4. Discussion

Most previous studies on repurposing HDTs for TB have largely focused on identifying drugs that target known disease-associated genes [21], [22], [23], [24], not disease-drug transcriptomic reversal, often overlooking the broader host response that can be captured by transcriptomic signatures. In contrast, our study brings connectivity-based transcriptomic methods into TB drug repurposing, leveraging disease-drug reversal at the level of differential gene expression to prioritize FDA-approved drugs that modulate the host transcriptional response to infection.

### 4.1 Summary of key findings

We developed an integrative drug repurposing workflow leveraging multiple TB host transcriptomics studies across various biological and technical conditions and incorporating multiple connectivity-based methods [7] to identify candidate host-directed therapeutics for TB. Our approach further implemented a novel signature aggregation method to address heterogeneity across disease datasets, generating a consensus disease signature for disease-drug signature comparison. This approach successfully recovered several known TB-relevant HDTs, such as statins and calcitriol, and also prioritized novel candidates including nelfinavir — a well-established HIV-1 protease inhibitor structurally and functionally similar to saquinavir — suggesting potential to restore lysosomal protease activity in TB-infected macrophages and enhance host immune responses (Table 1; Table S2) [74]. The top predicted HDTs were enriched for diverse host-targeted mechanisms of action, including HMGCR inhibition, vitamin D receptor activation, STAT pathway interference, HIV protease inhibition, and modulation of estrogen and dopamine signaling (Table 2). These mechanisms reflect distinct strategies for promoting autophagy, enhancing antimicrobial activity, and modulating host immunity, offering promising directions for further investigation and therapeutic development.

### 4.2 Advancing transcriptome-based drug repurposing for infectious diseases

While transcriptome-based drug repurposing has been widely applied in genetic diseases and some viral infections like COVID-19, it remains underused in TB and other bacterial infections. Most prior TB efforts have relied on structure- or target-based methods such as molecular docking, systems pharmacology, network inference, or pathway enrichment to identify host-directed candidates [21], [22], [23], [24]. These approaches often depend on predefined disease- and drug-associated genes and do not fully capture the dynamic nature of host transcriptional responses to infection. Although some studies have defined TB gene signatures, they are mostly used for diagnostics, not therapeutic discovery [25]. The limited use of transcriptome reversal strategies in TB represents a missed opportunity to prioritize drugs based on how well they restore infection-altered host expression toward a healthy state.

Our work fills this gap by applying a transcriptome-based drug repurposing framework for TB. Instead of targeting individual genes, we focus on reversing the overall disease signature using multiple connectivity metrics across diverse TB datasets. This allows us to capture dominant TB host responses and prioritize compounds that shift those responses toward a healthier state. By recovering known HDTs and identifying novel candidates, our approach demonstrates the value of integrating transcriptomic reversal into TB drug repurposing and expands the methods for host-directed therapy development in infectious diseases.

### 4.3 Methodological innovations and their impact

Transcriptomic responses to TB infection vary substantially across datasets due to variety in profiling technologies, technical and biological conditions. Our workflow navigates this heterogeneity by incorporating multiple individual disease signatures and aggregated (consensus) signatures. We demonstrated that our weighted average aggregation strategy effectively captures the diverse disease signals at the pathway level observed across the individual signatures (Figure 2). Furthermore, the drug prioritization results showed strong agreement between individual and aggregated signatures, reinforcing confidence in the final predictions with 140 out of 160 predicted drugs shared across the two sets (Table S2). These findings highlight the importance of summarizing disease signals across multiple signatures rather than relying on a single signature. By extracting dominant transcriptional signals, our approach uncovers core host response patterns that are critical for identifying effective disease-drug reversal candidates, even in the presence of underlying heterogeneity.

In addition to signature aggregation, we implemented six complementary methods, four enrichment-based (CMAP 1.0, LINCS: WCS, NCS, Tau) and two correlation-based (XCor, XSpe), to quantify drug reversal potential. These methods capture distinct aspects of disease-drug relationships, including gene distribution patterns, gene membership, and ranked or unranked associations. Due to their differing mathematical foundations, each method introduces unique biases, leading to variability in drug rankings. These biases are reflected in the tendency of drugs to cluster within methods of the same category (Table S2), emphasizing the modality-specific nature of the scoring results. To manage variability across scoring methods, we applied a rank-based aggregation strategy that respects modality-specific scoring differences to prioritize drugs consistently highly ranked across methods, increasing the stability and interpretability of the final drug list. Using multiple connectivity metrics across scoring modalities and combining them with rank aggregation accounts for method-specific biases and strengthens confidence in predicted drug candidates. The success of this integration is reflected in known TB-relevant HDTs such as statins and calcitriol consistently ranked highly across multiple methods (Table 1). Additionally, network analysis provides mechanistic insights into disease-drug relationships by uncovering shared pathways (Figure S3) and highlighting potential therapeutic gene targets based on their connectivity within the PPI network (Figure 3).

### 4.4 Limitations and technical considerations

A major challenge in using the LINCS drug perturbation dataset (Methods 2.2) is the variation across drug signatures due to differences in cell lines, dosages, and treatment durations. For consistency, we restricted our analysis to a uniform condition of 10 μM concentration with a 24-hour treatment and summarized drug effects in a cell-line-agnostic manner (Methods 2.6). Despite using this single drug condition, the limited diversity and incomplete coverage of cell types within LINCS remain a limitation. To explore potential biological matches for disease-drug reversal analysis, we assessed the similarity between healthy control samples from our disease datasets and untreated LINCS cell lines (Methods 2.12). Pearson correlation, selected based on its consistency with other methods in recovering the top-matching cell line, revealed some expected biological groupings, particularly among blood-derived and hematopoietic/lymphoid samples (Figure S4a). However, correlation values were consistently near zero across different metrics, limiting our ability to define confident baseline matches and underscoring the need for more systematic strategies for evaluating biologically relevant baselines (Figure S4b).

To ensure compatibility with the drug perturbation data, we restricted our disease-drug reversal scoring to the 978 landmark genes defined by the L1000 assay [97] — a representative gene set whose directly measured expression profiles enable robust transcriptome inference. While this constraint improves consistency across datasets and avoids the sparsity and noise associated with inferred gene values, it reduces overall gene coverage and may exclude disease-associated genes not captured in the landmark set. Future work could explore incorporating inferred genes with confidence weighting or validating findings using transcriptome-wide data sources, such as RNAseq-like drug profiles (e.g., CycleGAN-based predictions [98]). Ultimately, experimental validation will be necessary to confirm the therapeutic efficacy of our prioritized TB HDT candidates, as single drugs or synergistic combinations.

### 4.5 Therapeutic implications and future directions

Our approach effectively identified both known and novel HDT candidates for tuberculosis, highlighting its broader use for HDT drug repurposing in other infectious diseases. Our MOA-level synergy analysis provides a framework for informing rational design of combination therapies, offering insights into which drug pairs may act synergistically based on complementary or convergent MOAs. Antagonistic MOA pairs showing strong antagonism by our analysis may help flag drug combinations to avoid—especially in patients with comorbidities or those on long-term medications. Moreover, network-based analysis of our predicted HDTs revealed novel druggable targets that mediate interactions between disease genes in our aggregated signatures and drug-target genes within the PPI network. This systems-level understanding of disease-drug connectivity strengthens the rationale for targeting specific host pathways and further supports the identification of combinatorial therapeutics. These findings provide a solid starting point for experimental validation, taking us from computational predictions to actual testing in vitro and in vivo to assess their therapeutic efficacy. Future work should also incorporate drug cell-line contexts into the predictions, helping identify the most relevant cell lines for validating these targets and drug combinations. This approach has the potential to accelerate the development of more effective HDT strategies, not only for tuberculosis but also for a wide range of infectious diseases.

## 5. Conclusion

We present a computational framework that integrates heterogeneous host transcriptomic datasets and multiple connectivity-based scoring schemes to identify host-directed therapeutic candidates for TB. While heterogeneity in biological and technical conditions across datasets remains a challenge, our approach is specifically designed to prioritize robust HDT predictions despite such variability. By aggregating connectivity scores and weighting disease differential expression profiles based on similarity, we identify drug candidates that consistently reverse TB-associated gene signature patterns across platforms, studies, and technologies.

Our results demonstrate the utility of this integrative strategy: we recovered known HDTs, uncovered promising mechanisms of action, and identified druggable gene targets and potential synergistic combinations supported by gene-gene interaction networks and current literature. This framework not only advances TB therapeutic discovery but also provides a scalable scheme for applying transcriptome-based drug repurposing to other infectious diseases.

## Supplementary Material

Supplement 1

## Figures and Tables

**Table 1.** Top 20 high-confidence drug candidates with supporting literature evidence. Drug candidates were prioritized based on their mean rank scores, which reflect the overall ranking of each drug across predictions from both individual and aggregated TB signatures using six complementary connectivity scoring methods. Shown are the top 20 ranked drug candidates, along with literature evidence supporting their potential as host-directed therapeutics against TB. “No evidence” indicates no direct published support for the drug’s use in TB. See Table S2 for the full list of predicted candidates.

Drug candidate	Mean rank score	Literature evidence
atorvastatin	0.81	Adewole et al., 2023 [60]
calcitriol	0.80	Wallis and Zumla, 2016 [61]
niclosamide	0.74	Fan et al., 2019 [62]
nelfinavir	0.72	No prior evidence
fluphenazine	0.69	Heemskerk et al., 2021 [63]
vemurafenib	0.59	No prior evidence
fluvastatin	0.59	Montero-Vega et al., 2024 [56]
tamoxifen	0.58	Boland et al., 2023 [4]
lovastatin	0.55	Jhilta et al., 2025 [57]
rosuvastatin	0.54	Cross et al., 2024 [58]
digitoxin	0.52	Magalhães et al., 2025 [64]
chloroxine	0.50	Zhao et al., 2023 [65]
fostamatinib	0.49	Ponnusamy et al., 2022 [24]
hexylresorcinol	0.48	Nikolaev et al., 2024 [66]
clomifene	0.48	Lee et al., 2021 [67]
crizotinib	0.48	No prior evidence
digoxin	0.46	Magalhães et al., 2025 [64]
ouabain	0.45	No prior evidence
phenazopyridine	0.44	No prior evidence
chlorpromazine	0.43	Viveiros et al., 2001 [68]

**Table 2.** Mechanisms of action (MOAs) associated with predicted drug results, with literature evidence. MOAs linked to top-ranked drug candidates with literature evidence describing their relevance to TB infection or host-directed mechanisms. These include inhibition of cholesterol synthesis, modulation of immune pathways (e.g., vitamin D and STAT signaling), enhancement of macrophage antimicrobial activity, and activation of autophagy. References highlight prior studies supporting the therapeutic relevance of these MOAs against *Mycobacterium tuberculosis*.

Top MOAs associated with predicted drug candidates	Literature evidence against TB infection	Reference
HMGCR inhibitor	Mycobacterium tuberculosis uses cholesterol in the host macrophage to infect and survive inside the host macrophage. Statins inhibit the synthesis of cholesterol and aid in reducing the levels of cholesterol in humans.	Su et al., 2021; Young et al., 2020 [69], [70]
Vitamin D receptor agonist	Vitamin D appears to play a major role in both the innate and adaptive immune systems • by enhancing the formation of autophagosomes to stimulate the expression of antimicrobial proteins. • by stimulating the development of suppressive regulatory T-cells while suppressing the development of inflammatory Th17 cells.	Chun et al., 2011 [71]
DNA replication receptor | STAT inhibitor	Targeting the DNA replication machinery in *M. tuberculosis*, specifically binding the DNA gyrase “receptor”, can block bacterial DNA synthesis. STAT3 inhibitors block IL-10-mediated immune suppression, restoring macrophage antimicrobial function against TB.	Ditse et al, 2017; Queval et al., 2016 [72], [73]
HIV protease inhibitor	The HIV protease inhibitor saquinavir restores cathepsin S activity suppressed by Mtb, enhancing antigen processing and HLA class II-mediated immune responses.	Pires et al., 2021 [74]
Dopamine receptor antagonist	Dopamine receptor genes showed decreased expression in patients with tuberculosis compared to normal individuals. This shows the potential of dopamine receptor antagonists to activate the autophagy pathway to kill TB bacteria.	Sheikhpour et al., 2021 [75]
RAF inhibitor	Inhibiting host RKIP with agents like locostatin may counteract the immunosuppressive effects of the Mtb protein Rv2140c, which suppresses ERK and NF-κB signaling to promote bacterial survival in macrophages.	Abo-Kadoum et al., 2021 [76]
Estrogen receptor antagonist | SERM	Selective estrogen receptor modulator (SERM) was also shown to inhibit intracellular *Mtb* growth in macrophages through enhanced ROS-dependent autophagy and to inhibit Mtb growth in liquid culture.	Ouyang et al., 2020 [77]

**Table 3.** Identified potential druggable genes with literature evidence. Summary of key host genes identified in the disease-drug-target network as mechanistic bridges (in-path genes) connecting TB-perturbed genes and known drug targets. Each gene listed is supported by literature evidence for its involvement in host immune response or cellular processes relevant to TB infection. These genes may serve as potential targets for host-directed TB therapeutics.

Identified in-path genes	Literature evidence against TB infection	Reference
MYD88	The adapter protein Myd88 plays an important role in limiting mycobacterial growth in a zebrafish model for tuberculosis. Myd88 fosters bacterial containment and is necessary to raise an adequate innate and acquired immune response to *Mycobacterium tuberculosis* (*Mtb*).	Hosseini et al., 2021; Cervantes 2017 [79], [80]
RELA	More on the pathogen side — *M. tuberculosis* has a single relA homolog (relMtb) responsible for initiating the stringent response. Deleting relMtb makes the bacterium less able to survive nutrient deprivation and persist in a mammalian host.	Dahl et al., 2005 [81]
CXCR2	CXCR1/CXCR2 may be important for both cellular recruitment and mycobacterial killing in vitro.	Slight and Khader 2013 [82]
UBC	A surface protein on Mtb, Rv1468c, directly binds to host ubiquitin, initiating a selective autophagy process called xenophagy. This interaction, independent of ubiquitin ligases, recruits the autophagy receptor p62, leading to the engulfment and degradation of Mtb.	Chai et al., 2019 [83]
GRB2	The MR-Grb2-SHP-1 signaling axis plays a significant role in the host response to TB. While this pathway is involved in the initial phagocytosis of Mtb, the bacteria exploit this pathway to inhibit phagosome maturation, enabling their survival and growth within macrophages.	Rajaram et al, 2017 [84]
CBL	Mtb may hijack the FAF2 pathway to impair host defenses, and CBL acts to constrain FAF2 activity in infected macrophages.	Truong et al., 2024 [85]
TIMP1	The relatively low levels of TIMP-1 in TB infection may allow for unchecked MMP activity, contributing to tissue damage and disease progression.	Friedland et al., 2002 [86]
TRAF6	TRAF6 plays a critical role in the host immune response to Mtb infection by promoting autophagy and the recruitment of immune cells to the site of infection.	Ma et al., 2023 [87]
IL8	Elevated IL-8 concentrations have been found in the bronchoalveolar lavage fluid, pleural fluid, and plasma of TB patients, as well as in M. tuberculosis-infected human tissue.1 In vivo studies have shown that pretreatment with anti-IL-8 alone inhibits mycobacterial granuloma formation, an important mechanism of host defense against TB.	Ameixa et al., 2002 [88]
TP53	p53, directly regulated by TP53, particularly within M1 macrophages, promotes apoptosis and reduces intracellular Mtb survival through the TLR2/JNK signaling pathway, leading to the production of reactive oxygen species and nitric oxide. The study using p53-deficient mice and human macrophages from tuberculosis patients suggests that enhancing p53 activity could be a novel therapeutic approach for tuberculosis.	Lim et al., 2020 [89]
