# Supplementary material for: Integrative transcriptome-based drug repurposing in tuberculosis
Source: bioRxiv. 2025 Jun 2:2025.06.02.657296. Preprint. [Version 1] doi: 10.1101/2025.06.02.657296 (PMC12157595; doi:10.1101/2025.06.02.657296)
Supplement: Supplement 1 [file media-1.pdf]

Integrative transcriptome-based drug repurposing in tuberculosis

Kewalin Samart[1,4], Landon Buskirk[2,4], Amy Tonielli[3,4], Arjun Krishnan[4*], Janani Ravi[4*].
[1]Computational Bioscience Program, School of Medicine, University of Colorado Anschutz Medical Campus, Aurora, CO, USA, [2]Data Science, Michigan State University, East Lansing, MI, USA, [3]Biomedical Laboratory Science, Michigan State University, East Lansing, MI, USA, [4]Department of Biomedical Informatics, Center for Health Artificial Intelligence, University of Colorado Anschutz Medical Campus, Aurora, CO, USA.

*Corresponding authors: arjun.krishnan@cuanschutz.edu, janani.ravi@cuanschutz.edu

Supplementary information

1. Additional materials and methods

1.1 Baseline comparison

To best determine the most biologically compatible pairs of expression baselines for disease-drug signature comparison, we assessed similarity between each of control disease samples and drug samples using Pearson, Spearman [1], Rank Biased Overlap (RBO) [2], and least absolute shrinkage and selection operator (LASSO) [3] approaches.

1.1.1 Data collection and preprocessing

The drug control samples i.e., gene expression of untreated cell lines were obtained from LINCS level 3 GSE92742 [4]. We excluded expression of non-landmark genes, therefore, only included 978 landmark genes for the analyses. We corrected the drug data distribution by performing a quantile normalization on all the drug control samples. Then, a 'target drug profile' was randomly sampled from the normalized drug data and used as the reference vector for the control disease data distribution mapping using quantile transformation; separately applied on the microarray and RNAseq data.

1.1.2 Baseline similarity assessment

Computation of summarized-similarity coefficients of each pairwise disease-drug control samples was calculated using the following metrics:

1. Pearson and Spearman correlation

Pearson and Spearman correlation assess how strong the linear relationship between a pair of drug and disease baselines is. While Pearson only considers the overall agreement trend of genes based on their expression values by looking at how well each gene aligns with their respective sample mean regardless of direction, Spearman correlation is a directional metric that takes into account the difference in ranks of the same gene from two baseline samples.

2. Rank-Biased Overlap (RBO)

Unlike Pearson and Spearman, which require two complete lists for comparison, RBO is a ranked-based measure with weight assignments to all the genes in each baseline sample. The genes ranked toward the top of the list based on absolute regulation level (highly up- or downregulated) get higher weights meaning if a gene is ranked the same or very close toward the top, then it would upweight the RBO metric. Overall, RBO is claimed to be suitable for feature selection as RBO coefficients are low for the genes with a large difference in

ranks, similar to zeroing out unimportant features. Therefore, RBO metric contribution only considers genes with a high rank agreement between two lists.

3. Lasso coefficients

We adapted the concept of *SampleLasso* [5] to quantify baseline similarity using L1-regularized regression. For each disease control sample, we trained a Lasso model to predict its expression profile as a sparse linear combination of the drug control cell line profiles. In modeling terms, the cell line profiles served as features, while each disease sample became a target. The resulting Lasso coefficients, by representing the contribution of each cell line profile to reconstructing the disease profile, were used as our similarity metric.

2. Supplementary Figures

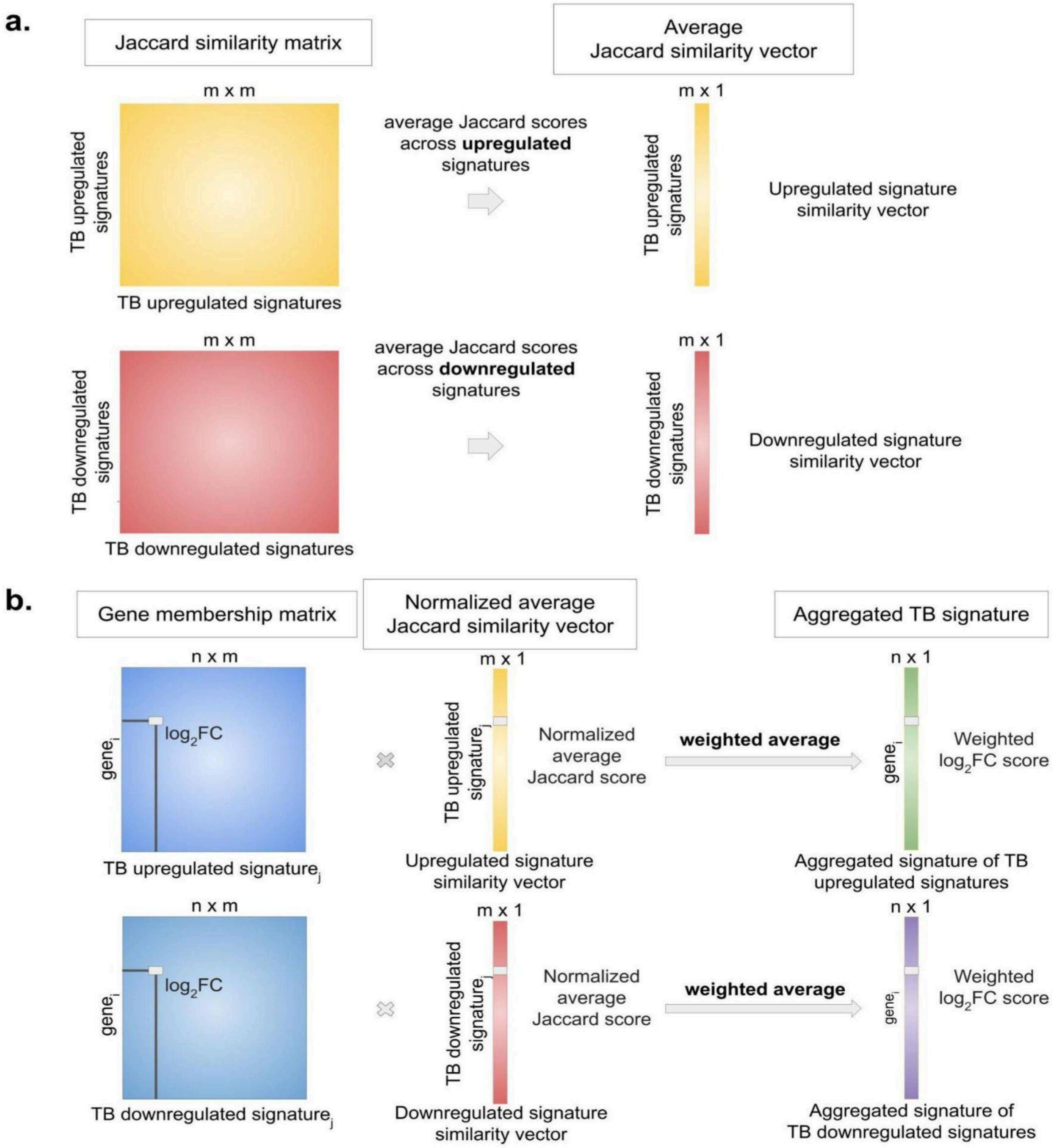

Figure S1. Signature aggregation scheme.
(a) Overview of the similarity-weighting step used to assign weight a proxy of confidence or 'trust' to each individual TB signature. A Jaccard similarity matrix is computed across all pairwise combinations of upregulated (top) and downregulated (bottom) TB signatures. The average *Jaccard* score for each signature is then used to create a similarity vector representing its agreement with the rest of the group. **(b)** Construction of the aggregated TB signature. Each gene's $\log_2$ fold change (differential expression) across individual signatures is combined using a weighted average, where weights are derived from the normalized *Jaccard* similarity vector. This process generates robust aggregated signatures for both up- and downregulated signature sets that emphasize consistent transcriptomic signals across studies.

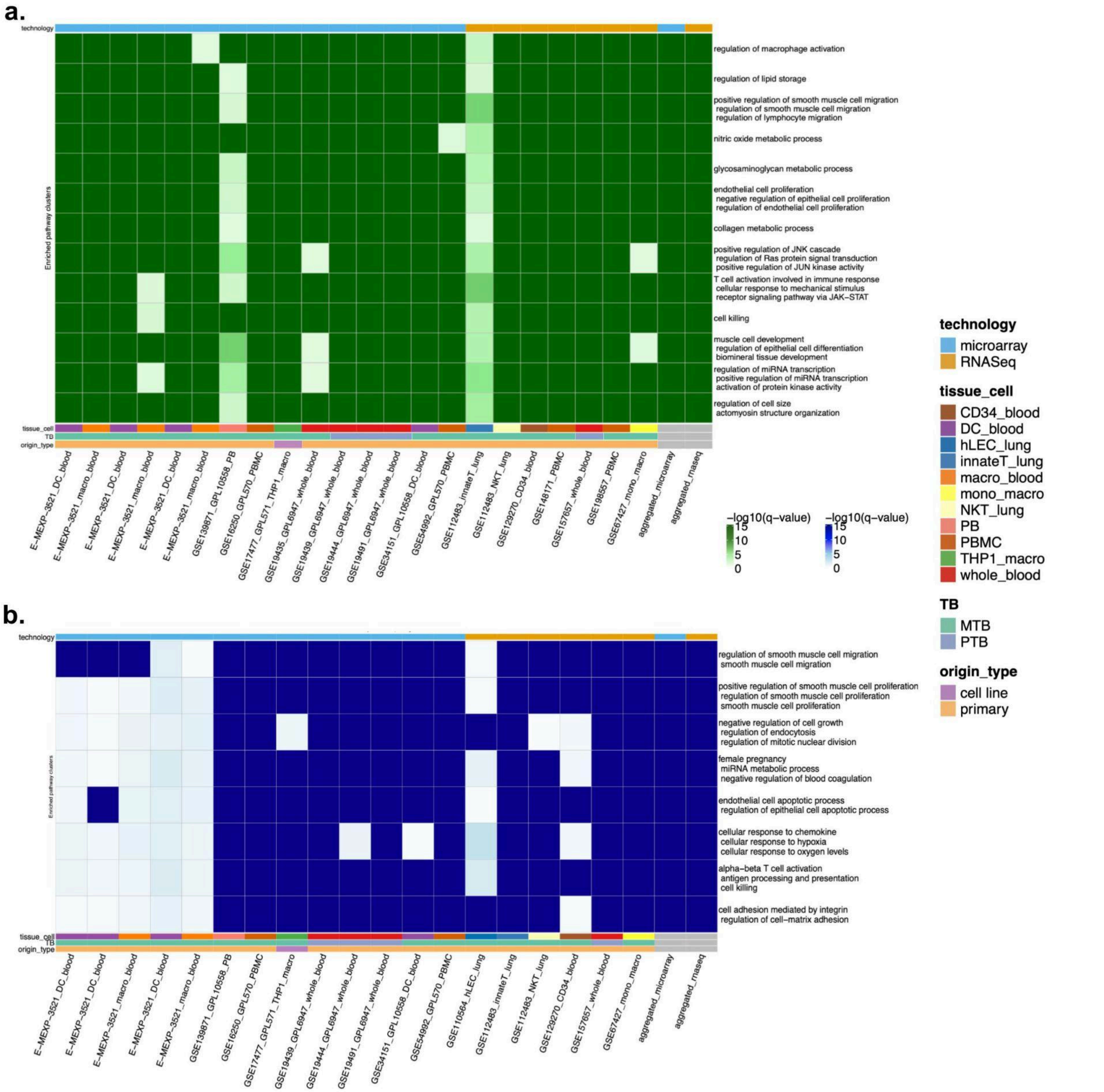

Figure S2. Aggregated signatures are enriched in pathway clusters summarizing pathway enrichment across individual signatures.

(a) Heatmap showing representative pathway clusters enriched across individual upregulated signatures and captured by the aggregated upregulated TB signature. **(b)** Heatmap showing pathway clusters present across individual downregulated signatures and represented in the aggregated downregulated TB signature. Each row represents a pathway cluster labeled by up to three GO:BP terms. Columns represent individual TB signatures, annotated by profiling technology, tissue/cell type, TB sample type (PTB: pulmonary; MTB: non-specified), and sample source (primary sample or cell line). Color intensity reflects pathway enrichment, with darker shades indicating stronger significance (higher –$\log_{10}$(q-value)).

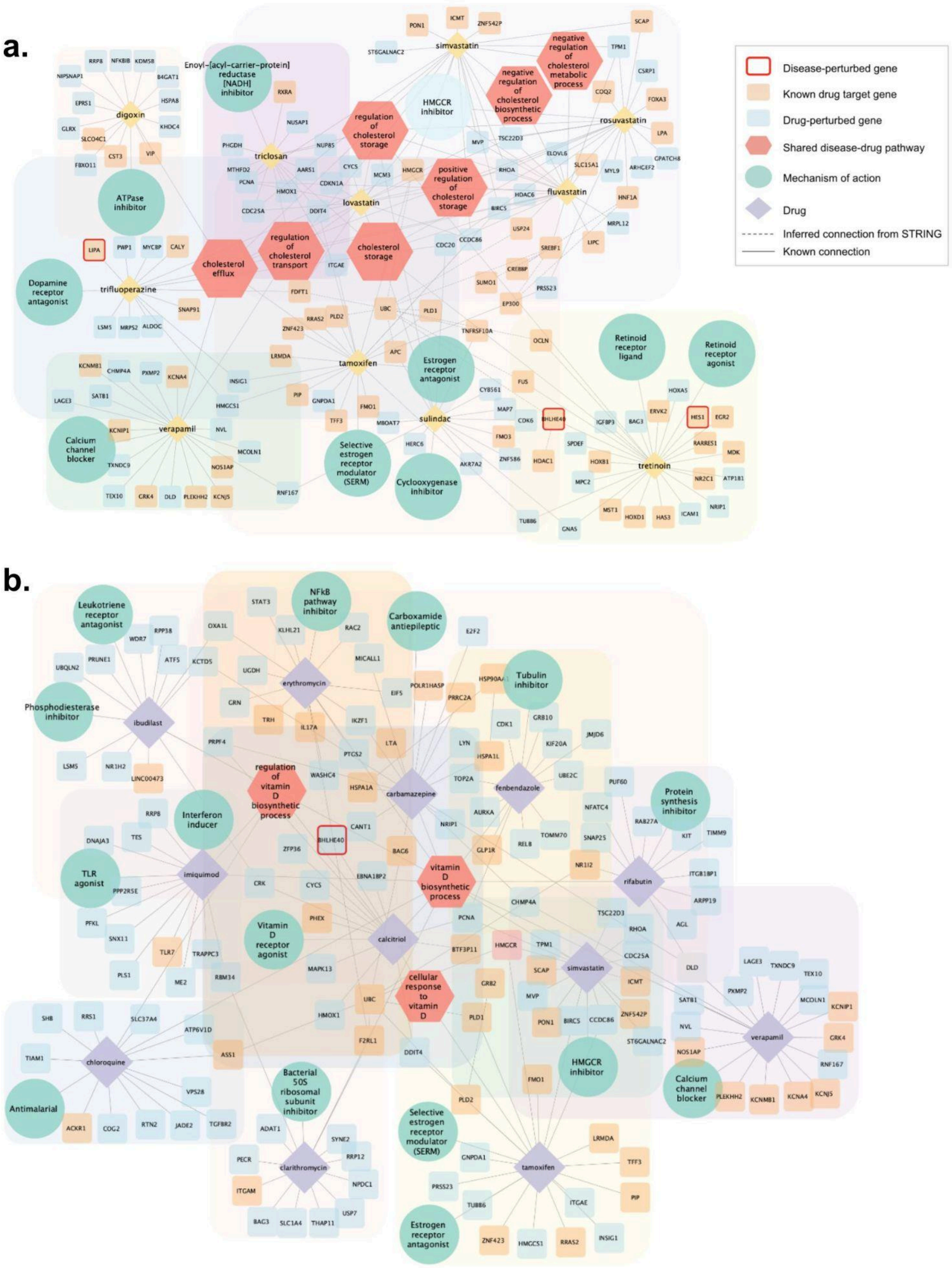
a.
Disease-perturbed gene
Known drug target gene
Drug-perturbed gene
Shared disease-drug pathway
Mechanism of action
Drug
Inferred connection from STRING
Known connection
Enoyl-[acyl-carrier-protein] reductase [NADH] inhibitor
HMGCR inhibitor
ATPase inhibitor
Dopamine receptor antagonist
Calcium channel blocker
Estrogen receptor antagonist
Selective estrogen receptor modulator (SERM)
Cyclooxygenase inhibitor
Retinoid receptor ligand
Retinoid receptor agonist
negative regulation of cholesterol metabolic process
negative regulation of cholesterol biosynthetic process
regulation of cholesterol storage
positive regulation of cholesterol storage
regulation of cholesterol transport
cholesterol storage
cholesterol efflux
simvastatin
rosuvastatin
fluvastatin
lovastatin
digoxin
triclosan
trifluoperazine
verapamil
tamoxifen
sulindac
tretinoin
b.
Leukotriene receptor antagonist
Phosphodiesterase inhibitor
NFkB pathway inhibitor
Carboxamide antiepileptic
Tubulin inhibitor
Protein synthesis inhibitor
Interferon inducer
TLR agonist
Vitamin D receptor agonist
Antimalarial
Bacterial 50S ribosomal subunit inhibitor
Selective estrogen receptor modulator (SERM)
Estrogen receptor antagonist
HMGCR inhibitor
Calcium channel blocker
regulation of vitamin D biosynthetic process
vitamin D biosynthetic process
cellular response to vitamin D
ibudilast
erythromycin
carbamazepine
fenbendazole
rifabutin
imiquimod
calcitriol
simvastatin
verapamil
chloroquine
clarithromycin
tamoxifen

Figure S3. Cholesterol- and vitamin D-related disease-drug pathway subnetworks.
(a) Subnetwork centered around cholesterol metabolism, showing interactions among TB disease-perturbed genes in our aggregated disease signatures (red outline), known drug targets (orange), and key shared pathway genes (peach) identified from shortest paths in the STRING protein–protein interaction (STRING-PPI) network. Several drugs and their mechanisms of action (green) converge on cholesterol-related processes, including HMGCR inhibitors and ATPase inhibitors. **(b)** Subnetwork centered on vitamin D–related immune regulation, highlighting shared genes across disease and drug mechanisms, including vitamin D receptor agonists, interferon inducers, and NF-κB pathway inhibitors. Both subnetworks illustrate connections between disease genes and predicted drugs via key intermediate nodes with high betweenness centrality, supporting mechanistic relevance of these pathways in TB infection and treatment response. Solid lines represent known interactions from DGIdb; dashed lines indicate inferred connections from the STRING-PPI network.

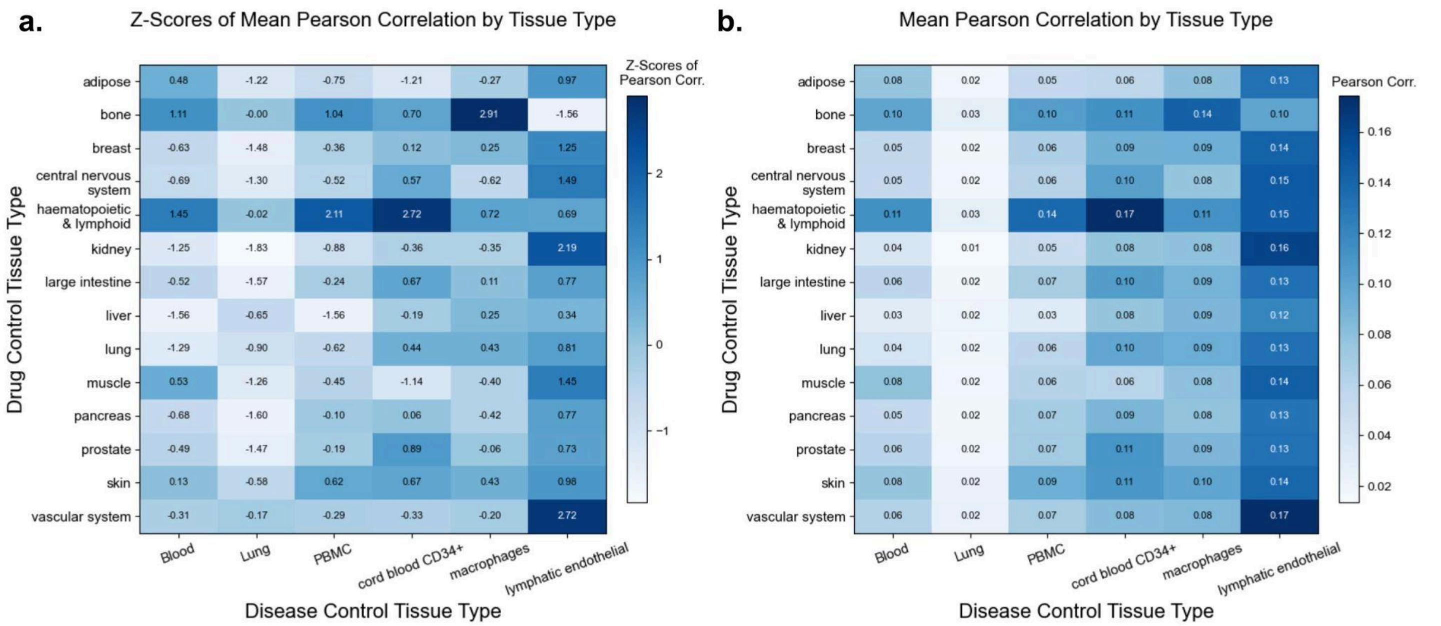

Figure S4. Pearson correlation of baseline disease-drug samples by tissue types.

(a) Heatmap of Z-scores from mean Pearson correlations between healthy control samples from TB disease datasets (columns) and untreated LINCS drug cell line profiles (rows), grouped by tissue type. Some biologically plausible groupings were observed, especially among blood and hematopoietic/lymphoid tissues.

(b) Heatmap of raw mean Pearson correlation values. Despite detectable patterns, overall correlation values were consistently low, limiting the ability to confidently define baseline-matched tissue pairs. These results highlight the need for improved methods to systematically evaluate biologically relevant baselines for disease-drug signature comparison

3. Supplementary Tables

Table S1. List of public TB datasets used in this work.

This table summarizes the metadata for TB gene expression datasets included in our analysis, grouped by profiling technology (microarray or RNA-seq). For each signature, we list the associated study ID, platform, TB status (MTB or PTB), tissue of origin (circulating vs. lung), origin type (primary vs. cell line), and cell or tissue type. The number of up- and downregulated genes represents differentially expressed genes used to construct disease signatures. Aggregated signatures represent consensus profiles generated across all microarray or RNA-seq studies, respectively.

TB Signature Metadata<br>Grouped by Technology								
Signature	Study	Platform	TB Status	Tissue Origin	Origin Type	Cell/Tissue Type	Up Genes	Down Genes
microarray								
E-MEXP-3521_NA_MTB18hD_control	E-MEXP-3521	-	MTB	circulating	primary	dendritic cell in blood	442	426
E-MEXP-3521_NA_MTB18hM_control	E-MEXP-3521	-	MTB	circulating	primary	macrophage in blood	477	395
E-MEXP-3521_NA_MTB48hD_control	E-MEXP-3521	-	MTB	circulating	primary	dendritic cell in blood	424	475
E-MEXP-3521_NA_MTB48hM_control	E-MEXP-3521	-	MTB	circulating	primary	macrophage in blood	466	407
E-MEXP-3521_NA_MTB4hD_control	E-MEXP-3521	-	MTB	circulating	primary	dendritic cell in blood	521	345
E-MEXP-3521_NA_MTB4hM_control	E-MEXP-3521	-	MTB	circulating	primary	macrophage in blood	516	356
GSE139871_GPL10558_MTB_control	GSE139871	GPL10558	MTB	circulating	primary	peripheral blood	406	533
GSE16250_GPL570_MTB_control	GSE16250	GPL570	MTB	circulating	primary	peripheral blood mononuclear cell	537	308
GSE17477_GPL571_MTB_control	GSE17477	GPL571	MTB	circulating	cell line	thp-1 macrophage	347	547
GSE19435_GPL6947_MTB_control	GSE19435	GPL6947	MTB	circulating	primary	whole blood	479	341
GSE19439_GPL6947_PTB_control	GSE19439	GPL6947	PTB	circulating	primary	whole blood	308	506
GSE19444_GPL6947_PTB_control	GSE19444	GPL6947	PTB	circulating	primary	whole blood	197	648
GSE19491_GPL6947_PTB_control	GSE19491	GPL6947	PTB	circulating	primary	whole blood	230	626
GSE29536_GPL10558_PTB_control	GSE29536	GPL10558	PTB	circulating	primary	whole blood	575	265
GSE34151_GPL10558_MTB_control	GSE34151	GPL10558	MTB	circulating	primary	dendritic cell in blood	473	484
GSE54992_GPL570_MTB_control	GSE54992	GPL570	MTB	circulating	primary	PBMCs	446	464
aggregated_TB_signature1	-	-	-	-	-	-	87	63
RNASeq								
GSE110564_MTB_control	GSE110564	-	MTB	lung	primary	lymphatic endothelial cells (hLEC) in lung	36	99
GSE112483_MTBam_control	GSE112483	-	MTB	lung	primary	alveolar macrophages in lung	62	24
GSE112483_MTBmait_control	GSE112483	-	MTB	lung	primary	mucosal associated innate T cells in lung	10	3
GSE112483_MTBnkt_control	GSE112483	-	MTB	lung	primary	natural killer T cells in lung	16	10
GSE129270_MTB72h_control	GSE129270	-	MTB	circulating	primary	purified cord blood CD34+ cells	187	93
GSE148171_MTB_control	GSE148171	-	MTB	circulating	primary	PBMCs	172	53
GSE157657_PTB_control	GSE157657	-	PTB	circulating	primary	whole blood	265	263
GSE198557_MTB_control	GSE198557	-	MTB	circulating	primary	PBMCs	112	12
GSE67427_MTBrv18h_control	GSE67427	-	MTB	lung	primary	monocyte-derived macrophages	80	58
aggregated_TB_signature2	-	-	-	-	-	-	97	17

Table S2. Full list of 140 high confidence drug candidates with score-level support across microarray and RNA-seq signatures.

Each cell shows the number of individual TB signatures (out of 16 for microarray or 9 for RNA-seq) for which a given drug achieved a strong reversal score (i.e., within the top 10% most negative values) under each connectivity metric. The heatmap is split by scoring subcategories: CMAP 1.0, LINCS (NCS, Tau, WCS), and correlation-based methods (Pearson, Spearman). Warmer colors (red) represent results from microarray TB signatures; cooler colors (blue) represent RNAseq signatures. Rows correspond to 140 high-confidence predicted TB HDT candidates that appeared in both individual and aggregated analyses, and are ranked by their overall mean rank score. This visualization highlights which drugs are consistently supported across signatures and metrics, reinforcing their prioritization strength.

		Microarray (16 signatures)						RNAseq (9 signatures)					
		Enrichment-based				Correlation-based		Enrichment-based				Correlation-based	
		CMAP1.0	LINCS			Correlation		CMAP1.0	LINCS			Correlation	
	MeanRank	microarray_CMAP	microarray_NCS	microarray_Tau	microarray_WCS	microarray_Cor_pearson	microarray_Cor_spearman	RNAseq_CMAP	RNAseq_NCS	RNAseq_Tau	RNAseq_WCS	RNAseq_Cor_pearson	RNAseq_Cor_spearman
atorvastatin	0.812437305	13	9	9	9	9	9	5	0	0	0	0	0
calcitriol	0.801658115	10	0	9	0	9	0	6	0	5	0	5	0
niclosamide	0.741165780	10	9	9	9	10	0	5	0	0	0	5	0
nelfinavir	0.723171675	10	13	10	11	9	0	6	0	0	0	0	0
fluphenazine	0.689599629	11	11	11	0	9	9	5	0	5	0	0	0
vemurafenib	0.594566150	9	0	0	0	0	0	5	0	0	0	5	0
fluvastatin	0.589829219	12	11	0	10	10	10	0	0	0	0	0	0
tamoxifen	0.582786303	12	10	10	0	0	0	0	5	5	0	0	5
lovastatin	0.549420471	11	12	0	11	10	9	0	0	0	0	0	0
rosuvastatin	0.535302400	12	12	10	10	10	11	0	0	0	0	0	0
digitoxin	0.517567652	10	10	10	9	9	9	0	0	0	0	0	0
chloroxine	0.500966970	9	0	0	0	0	0	0	5	5	0	0	0
fostamatinib	0.485720405	10	0	0	0	0	9	6	0	0	0	0	0
hexylresorcinol	0.484709086	0	0	0	0	0	0	5	0	0	0	0	0
clomifene	0.484384065	9	10	0	9	9	0	0	5	5	0	0	0
crizotinib	0.479293903	11	9	0	0	9	10	0	0	0	0	0	0
digoxin	0.455438325	10	10	0	0	9	10	0	0	0	0	0	0
ouabain	0.453593042	9	10	0	0	0	9	0	6	5	0	0	0
phenazopyridine	0.437802232	0	0	0	0	0	0	5	0	0	0	0	0
chlorpromazine	0.430309597	0	0	0	0	0	0	5	0	0	0	0	0
retinol	0.423562522	0	0	0	0	0	0	5	0	0	0	0	0
teniposide	0.420697753	9	11	10	10	0	0	0	0	0	0	0	0
fenbendazole	0.393762725	0	9	9	0	0	0	5	6	6	0	0	0
neratinib	0.359304408	9	0	0	0	0	0	0	0	0	0	0	0
methylene-blue	0.339242846	10	0	0	0	0	0	0	0	0	0	0	0
fluoxetine	0.331281116	0	11	9	10	0	0	0	0	0	0	0	0
podophyllotoxin	0.321758049	10	0	0	0	0	0	0	0	0	0	0	0
tretinoin	0.306058711	9	0	0	0	0	0	0	0	0	0	0	0
docetaxel	0.302883416	0	0	0	0	0	0	0	5	5	0	0	0
simvastatin	0.300183254	13	12	0	0	9	10	0	0	0	0	0	0
bromocriptine	0.290429544	0	10	10	9	0	0	0	0	0	0	0	0
vindesine	0.282467250	9	0	0	0	0	0	0	0	0	0	0	0
mycophenolic-acid	0.275221458	11	0	0	9	9	0	0	0	0	0	0	0
thiostrepton	0.275181823	10	0	0	0	0	0	0	0	0	0	0	0
perphenazine	0.270887929	14	12	0	0	0	0	0	0	0	0	0	0
irinotecan	0.269098152	0	12	10	9	0	0	0	0	0	0	0	0
amitriptyline	0.253407008	0	0	0	0	0	0	5	0	0	0	0	0
triflupromazine	0.231390609	0	0	0	0	0	0	5	5	0	0	0	0
thioproperazine	0.208311677	0	0	0	0	0	0	0	5	5	0	0	0
metolazone	0.198218604	0	0	0	0	0	0	0	5	6	0	0	0
mosapride	0.189873331	0	9	0	0	0	0	0	5	0	0	0	0
sulindac	0.188748890	0	0	0	0	0	0	5	0	0	0	0	0
dasatinib	0.186092847	0	0	0	0	9	9	5	0	0	0	5	0
nilutamide	0.186059203	0	0	0	0	0	0	0	5	5	0	0	0
triamterene	0.185801715	9	0	0	0	9	11	0	0	0	0	0	0
ivermectin	0.180982606	10	0	0	0	0	0	0	0	0	0	0	0
vorinostat	0.178551873	9	0	0	0	0	0	0	0	0	0	0	0
sertraline	0.163029271	9	9	9	0	0	0	0	0	0	0	0	0
prednicarbate	0.160474729	10	0	0	0	0	0	0	0	0	0	0	0
fasudil	0.150187650	0	0	0	0	0	0	5	0	0	0	0	0
clofarabine	0.136868067	0	10	9	0	0	0	0	0	0	0	0	0
triclosan	0.134716546	10	0	0	0	10	0	0	0	0	0	0	0
everolimus	0.133680221	9	10	9	0	0	0	0	0	0	0	0	0
imiquimod	0.112444777	0	0	0	0	0	0	0	5	5	0	0	0
guanfacine	0.109794197	0	0	0	0	0	0	0	5	6	0	0	0
piretanide	0.102176717	0	0	0	0	0	0	0	5	0	0	0	0
homoharringtonine	0.100642289	0	9	0	0	0	0	0	0	0	0	0	0
clozapine	0.100292568	0	9	9	0	9	0	0	5	0	0	0	0
loperamide	0.082905135	0	9	0	0	0	0	0	0	0	0	0	0
scopolamine	0.077078852	0	9	0	0	0	9	0	0	0	0	0	0
flupentixol	0.077033095	10	0	9	0	0	0	0	0	0	0	0	0
salmeterol	0.073351094	0	9	9	0	0	0	0	5	5	0	0	0
phenoxybenzamine	0.069944165	0	9	12	0	0	0	0	0	0	0	0	0
paclitaxel	0.059662067	0	0	0	0	0	0	0	5	0	0	0	0
betahistine	0.049658011	0	0	0	0	0	0	0	5	5	0	0	0
sorafenib	0.046251442	0	0	0	0	11	10	6	0	0	0	0	0
trifluoperazine	0.042859360	0	9	0	0	0	0	0	0	0	0	0	0
topotecan	0.016078862	0	0	0	0	0	0	0	5	0	0	0	0
bendroflumethiazide	0.009136018	0	0	0	0	0	0	0	5	6	0	0	0
clonidine	0.008253506	0	0	0	0	0	0	0	6	0	0	0	0
phentolamine	0.005168659	0	10	0	0	0	0	0	0	0	0	0	0

		Microarray (16 signatures)						RNAseq (9 signatures)					
		Enrichment-based				Correlation-based		Enrichment-based				Correlation-based	
		CMAP1.0	LINCS			Correlation		CMAP1.0	LINCS			Correlation	
	MeanRank	microarray_CMAP	microarray_NCS	microarray_Tau	microarray_WCS	microarray_Cor_pearson	microarray_Cor_spearman	RNAseq_CMAP	RNAseq_NCS	RNAseq_Tau	RNAseq_WCS	RNAseq_Cor_pearson	RNAseq_Cor_spearman
mepacrine	-0.007255367	0	0	0	0	0	0	0	5	0	0	0	0
forskolin	-0.011595447	0	0	11	0	9	0	0	0	0	0	0	0
verapamil	-0.013145066	0	0	0	0	0	0	0	5	0	0	0	0
menadione	-0.015757990	0	9	0	0	0	0	0	0	0	0	0	0
nitrendipine	-0.023782476	0	9	10	0	0	0	0	0	0	0	0	0
dipyridamole	-0.029233661	0	0	0	0	0	0	0	6	0	0	0	0
pyrvinium-pamoate	-0.035772215	0	9	0	0	0	0	0	0	0	0	0	0
equol	-0.041515209	0	0	11	0	0	0	0	0	0	0	0	0
sirolimus	-0.046013055	0	0	0	0	0	9	5	5	0	0	0	5
flunarizine	-0.053412737	0	0	0	0	0	0	0	5	0	0	0	0
mianserin	-0.059848280	0	0	10	0	0	0	0	0	0	0	0	0
niacin	-0.062605778	0	0	11	0	0	0	0	0	0	0	0	0
prochlorperazine	-0.067062140	0	0	0	0	0	0	0	5	0	0	0	0
homochlorcyclizine	-0.090146433	0	0	9	0	0	0	0	0	0	0	0	0
enalapril	-0.093456929	0	0	11	0	0	0	0	0	0	0	0	0
benzydamine	-0.095605356	0	0	0	0	0	0	0	5	0	0	0	0
maprotiline	-0.101691570	0	0	0	0	0	0	0	5	0	0	0	0
progesterone	-0.106484544	0	0	9	0	0	0	0	0	0	0	0	0
haloperidol	-0.117553412	0	0	10	0	0	0	0	0	0	0	0	0
lamotrigine	-0.128950206	0	0	10	0	0	0	0	0	0	0	0	0
minoxidil	-0.133226147	0	0	11	0	0	0	0	0	0	0	0	0
propafenone	-0.142437006	0	0	9	0	0	0	0	0	0	0	0	0
flubendazole	-0.163612337	0	0	0	0	0	0	0	5	0	0	0	0
biotin	-0.185230888	0	0	0	0	0	0	0	5	0	0	0	0
ramipril	-0.187821121	0	0	0	0	0	0	0	0	5	0	0	0
ubenimex	-0.194779465	0	0	0	0	0	0	0	0	5	0	0	0
indapamide	-0.214710395	0	0	9	0	0	0	0	5	5	0	0	0
estradiol	-0.230785061	0	0	9	0	0	0	0	6	7	0	0	0
trapidil	-0.243542527	0	0	10	0	0	0	0	0	0	0	0	0
yohimbine	-0.253663211	0	0	9	0	0	0	0	0	0	0	0	0
aniracetam	-0.254064822	0	0	9	0	0	0	0	0	0	0	0	0
bosutinib	-0.267155585	0	0	0	0	9	11	0	0	0	0	0	0
valsartan	-0.269393564	0	0	9	0	0	0	0	0	0	0	0	0
azasetron	-0.269922368	0	0	10	0	0	0	0	0	0	0	0	0
isradipine	-0.273624849	0	0	9	0	0	0	0	0	0	0	0	0
chloroquine	-0.275995341	0	0	0	0	0	0	0	0	5	0	0	0
methylergometrine	-0.288284326	0	0	9	0	0	0	0	0	0	0	0	0
toremifene	-0.291025388	0	0	11	0	0	0	0	5	5	0	0	0
mestranol	-0.296026509	0	0	9	0	0	0	0	0	0	0	0	0
anagrelide	-0.302950876	0	0	0	0	0	0	0	0	5	0	0	0
labetalol	-0.313404292	0	0	0	0	0	0	0	0	5	0	0	0
erythromycin	-0.340516987	0	0	9	0	0	0	0	0	5	0	0	0
duloxetine	-0.340826965	0	0	10	0	0	0	0	0	6	0	0	0
calcifediol	-0.348597798	0	0	0	0	0	9	0	0	0	0	0	0
noretynodrel	-0.363540310	0	0	0	0	0	0	0	0	5	0	0	0
ibudilast	-0.406904202	0	0	0	0	0	0	0	0	0	0	0	5
clarithromycin	-0.409507525	0	0	0	0	0	0	0	0	5	0	0	0
noscapine	-0.418780163	0	0	0	0	0	0	0	0	5	0	0	0
benzthiazide	-0.419324971	0	0	0	0	9	0	0	0	0	0	0	0
meclofenamic-acid	-0.423628253	0	0	0	0	0	0	0	0	5	0	0	0
febuxostat	-0.458701392	0	0	9	0	0	0	0	0	5	0	0	0
penfluridol	-0.467328658	0	0	0	0	0	0	0	0	0	0	0	5
isoxsuprine	-0.480244332	0	0	0	0	10	0	0	0	0	0	0	0
ticlopidine	-0.486711316	0	0	0	0	0	0	0	0	5	0	0	0
tivozanib	-0.517882859	0	0	0	0	10	0	0	0	0	0	0	0
mebendazole	-0.523256145	0	0	10	0	0	0	0	0	5	0	0	0
fipronil	-0.524416948	0	0	0	0	0	10	0	0	5	0	0	0
rifabutin	-0.535712046	0	0	0	0	0	0	0	0	5	0	0	0
raltitrexed	-0.536518394	0	0	0	0	10	0	0	0	0	0	0	0
vincristine	-0.546809694	0	0	0	0	0	0	0	0	0	0	5	0
mepyramine	-0.557707056	0	0	0	0	9	0	0	0	0	0	0	0
fulvestrant	-0.561808371	0	0	0	0	0	0	0	0	0	0	0	5
ingenol	-0.642784401	0	0	0	0	9	0	0	0	0	0	0	0
carbamazepine	-0.643671762	0	0	0	0	9	0	0	0	0	0	0	0
dactinomycin	-0.724190478	0	0	0	0	9	0	0	0	0	0	0	0
acetyl-farnesyl-cysteine	-0.731713646	0	0	0	0	10	0	0	0	0	0	0	0
cabergoline	-0.755112080	0	0	0	0	0	0	0	0	0	0	5	0
papaverine	-0.783603940	0	0	0	0	0	9	0	0	5	0	0	0
mefenamic-acid	-0.900968857	0	0	0	0	9	0	0	0	0	0	0	0

Table S3. Key differentiating GO biological process terms enriched in the E-MEXP-3521 platform compared to other microarray datasets.

Significant GO biological process terms identified by a Mann–Whitney U test comparing enrichment scores between E-MEXP-3521 and all other microarray platforms. Reported terms reflect nuclear structure organization (e.g., RNA localization to Cajal bodies, telomere regulation), proton transport, oxidative stress, and metabolic reprogramming. These transcriptional differences are likely driven by the unique time-dependent sampling design of the E-MEXP-3521 study, rather than technical platform effects. P-values and adjusted p-values are reported in scientific notation, rounded to two decimals.

E-MEXP-3521 vs. other microarray platforms | Significant GO Terms from The Mann-Whitney U Test

	Enrichment Results		
GO Term	Raw p-value	Adjusted p-value	statistic
NADP metabolic process	**4.26e-04**	**4.44e-02**	54
RNA localization to Cajal body	**4.26e-04**	**4.44e-02**	54
RNA localization to nucleus	**4.26e-04**	**4.44e-02**	54
positive regulation of establishment of protein localization to telomere	**4.26e-04**	**4.44e-02**	54
positive regulation of protein localization to Cajal body	**4.26e-04**	**4.44e-02**	54
positive regulation of protein localization to chromosome, telomeric region	**4.26e-04**	**4.44e-02**	54
positive regulation of telomerase RNA localization to Cajal body	**4.26e-04**	**4.44e-02**	54
protein localization to Cajal body	**4.26e-04**	**4.44e-02**	54
protein localization to chromosome, telomeric region	**4.26e-04**	**4.44e-02**	54
protein localization to nuclear body	**4.26e-04**	**4.44e-02**	54
proton transmembrane transport	**4.26e-04**	**4.44e-02**	54
regulation of establishment of protein localization to chromosome	**4.26e-04**	**4.44e-02**	54
regulation of establishment of protein localization to telomere	**4.26e-04**	**4.44e-02**	54
regulation of protein localization to Cajal body	**4.26e-04**	**4.44e-02**	54
regulation of protein localization to chromosome, telomeric region	**4.26e-04**	**4.44e-02**	54
regulation of superoxide anion generation	**4.26e-04**	**4.44e-02**	54
regulation of telomerase RNA localization to Cajal body	**4.26e-04**	**4.44e-02**	54
telomerase RNA localization	**4.26e-04**	**4.44e-02**	54
telomerase RNA localization to Cajal body	**4.26e-04**	**4.44e-02**	54

Table S4. Cell line–to–tissue mapping for LINCS drug control samples used in baseline comparisons. Each cell line used in the LINCS dataset for untreated drug control profiling was annotated with its corresponding tissue of origin. These mappings were used to assess baseline similarity between disease and drug expression profiles. Cell lines with unknown or ambiguous tissue were removed.

Cell_Line	Tissue
A375	skin
A549	lung
HCC515	lung
BT20	breast
HME1	breast
HS578T	breast
MCF10A	breast
MCF 7.00	breast
MDAMB231	breast
SKBR3	breast
HA1E	kidney
HELA	large intestine
HT29	large intestine
HEPG2	liver
HUVEC	vascular system
JURKAT	haematopoietic and lymphoid tissue
LNCAP	prostate
PC3	prostate
YAPC	pancreas
NPC	central nervous system
NPC.CAS9	central nervous system
NPC.TAK	central nervous system
ASC	adipose
ASC.C	adipose
CD34	bone
SKL	muscle
SKL.C	muscle

4. Supplementary references

[1] "Spearman Rank Correlation Coefficient," in *The Concise Encyclopedia of Statistics*, New York, NY: Springer New York, 2008, pp. 502–505. doi: 10.1007/978-0-387-32833-1_379.

[2] W. Webber, A. Moffat, and J. Zobel, "A similarity measure for indefinite rankings," *ACM Trans. Inf. Syst. TOIS*, vol. 28, no. 4, pp. 1–38, 2010, doi: 10.1145/1852102.1852106.

[3] R. Tibshirani, "Regression Shrinkage and Selection Via the Lasso," *J. R. Stat. Soc. Ser. B Stat. Methodol.*, vol. 58, no. 1, pp. 267–288, Jan. 1996, doi: 10.1111/j.2517-6161.1996.tb02080.x.

[4] A. Subramanian *et al.*, "A Next Generation Connectivity Map: L1000 Platform and the First 1,000,000 Profiles," *Cell*, vol. 171, no. 6, pp. 1437-1452.e17, Nov. 2017, doi: 10.1016/j.cell.2017.10.049.

[5] C. A. Mancuso, J. L. Canfield, D. Singla, and A. Krishnan, "A flexible, interpretable, and accurate approach for imputing the expression of unmeasured genes," *Nucleic Acids Res.*, vol. 48, no. 21, p. e125, Dec. 2020, doi: 10.1093/nar/gkaa
